# Single‐Cell Transcriptome Analysis Reveals That Hmga2 Regulates Neuroinflammation and Retinal Function by Modulating Müller Cell Autophagy Through PI3K/AKT Signaling Following MCAO‐Induced Retinal Ischemia

**DOI:** 10.1002/advs.202502534

**Published:** 2025-08-30

**Authors:** Weihao Lv, Juzheng Yuan, Zhe Ruan, Ya‐nan Dou, Hongchen Zhang, Xiaowei Fei, Leiying Chen, Zehan Zhang, Kai Yang, Xiuquan Wu, Zhou Fei, Fei Fei

**Affiliations:** ^1^ Department of Neurosurgery Xijing Hospital Fourth Military Medical University Xi'an Shaanxi 710032 China; ^2^ Department of Ophthalmology Xijing Hospital Fourth Military Medical University Xi'an Shaanxi 710032 China; ^3^ Department of General Surgery Xijing Hospital Fourth Military Medical University Xi'an Shaanxi Province China; ^4^ Department of Neurology Tangdu Hospital Fourth Military Medical University Xi'an Shaanxi 710032 China; ^5^ Department of General Surgery Tangdu Hospital Fourth Military Medical University Xi'an Shaanxi 710032 China

**Keywords:** autophagy, Hmga2, MCAO‐induced retinal ischemia, müller cells, neuroinflammation

## Abstract

Central retinal artery occlusion (CRAO) can lead to retinal ischemia (RI), resulting in painless vision impairment. Müller cells, the principal supporting glial cells of the retina, are distributed throughout its layers and play pivotal roles in maintaining retinal homeostasis by regulating inflammation, oxidative stress, and angiogenesis following RI. However, the specific role of Müller cells in RI induced by middle cerebral artery occlusion (MCAO) remains unclear. In this study, single‐nucleus RNA sequencing is employed to investigate transcriptional changes in various retinal cell types following MCAO‐induced RI. A novel Müller cell subpopulation, characterized by the highest expression of the high‐mobility group A2 gene (*Hmga2*), emerged after RI. Knockout of *Hmga2* alleviated neuroinflammation and RI‐related symptoms, potentially through binding to phosphoinositide 3‐kinase and regulating Müller cell autophagy. Based on these findings, a targeted strategy using hybrid nanoparticles composed of Müller cell membranes and liposomes—termed siRNA‐Hmga2@LMM—is developed to deliver siRNA against Hmga2. In vivo experiments revealed that intravitreal injection of siRNA‐Hmga2@LMM improved retinal function following RI. These findings provide new mechanistic insights and identify potential targets for the treatment of RI.

## Graphical abstract

Employing snRNA‐seq post‐MCAO‐induced retinal ischemia (RI), this study revealed a novel Hmga2‐high Müller cell subpopulation. Hmga2 knockout alleviated neuroinflammation and RI symptoms, potentially by binding PI3K and regulating Müller cell autophagy. Consequently, siRNA‐Hmga2@LMM nanoparticles, utilizing Müller cell membrane‐coated liposomes, are developed. Intravitreal siRNA‐Hmga2@LMM injection improved retinal function, offering new mechanistic insights and therapeutic targets for RI.

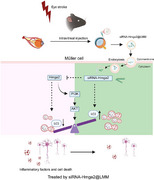

## 1 Introduction

Central retinal artery occlusion (CRAO), which leads to retinal ischemia (RI), is an acute, vision‐threatening ocular condition, with painless unilateral vision loss being the most common symptom.^[^ ^1^ ^]^ Epidemiological studies have indicated that the incidence of CRAO is ≈1 in 100 000 individuals.^[^ ^2^ ^]^ CRAO not only severely impacts daily life but also imposes significant psychological stress, thereby reducing patients’ quality of life. Unfortunately, current conservative treatments for CRAO, such as ocular massage and hyperbaric oxygen therapy, offer limited therapeutic benefits. Although thrombolytic therapy has demonstrated some efficacy, its application is restricted by a narrow therapeutic window—within 4.5 h of symptom onset.^[^ ^3^ ^]^ Clinical case reports have shown that some patients with acute ischemic stroke involving the internal carotid artery (ICA) also experience CRAO and subsequent RI with ipsilateral ocular visual impairment. This is due to the anatomical characteristic that the central retinal artery (CRA) is a terminal branch of the ophthalmic artery, which arises from the ICA.^[^ ^4^ ^]^ A similar RI phenomenon was observed in our previous study: after constructing a mouse model of middle cerebral artery occlusion (MCAO), some mice exhibited whitening of the ipsilateral eyeball, indicating RI.^[^ ^5^ ^]^

RI is a common pathological process underlying numerous ocular diseases, including glaucoma, diabetic retinopathy (DR), and CRAO. Substantial experimental evidence has demonstrated that RI triggers multiple pathological changes through mechanisms such as apoptosis, pyroptosis, ferroptosis, oxidative stress, autophagy, and inflammatory cascades. Müller glial cells, the predominant glial cell type in the retina—comprising 90% of retinal glial cells—extend radially across the entire thickness of the neural retina and serve as a supportive structure in the formation of both the outer and inner limiting membranes.^[^ ^6^ ^]^ Müller cells not only support the survival of photoreceptors and retinal ganglion cells (RGCs) while maintaining the morphology and physiological functions of the retina but also play critical roles in regulating immune and inflammatory responses^[^ ^7^ ^]^, serving as the primary responders to retinal injury. In DR, Müller cells modulate inflammation and angiogenesis through various signaling pathways.^[^ ^8^ ^]^ In vitro RI models have shown that reactive oxygen species production and inflammatory responses in Müller cells are key components of the pathological process.^[^ ^9^ ^]^ Moreover, it is indicated that Müller cell autophagy has been implicated in RI pathogenesis.^[^ ^10^ ^]^ Müller cells also contribute to the regulation of retinal angiogenesis following RI.^[^ ^11^ ^]^ In glaucoma models, activated Müller cells interact with microglia, resulting in upregulated expression of pro‐inflammatory cytokine mRNAs within the Müller cells.^[^ ^12^ ^]^ Furthermore, mineralocorticoid receptors in Müller cells have been linked to the pathogenesis of retinal macular edema.^[^ ^13^ ^]^ However, the specific role of Müller cells in RI induced by MCAO remains unclear.

The application of single‐cell sequencing technology has enabled high‐throughput analysis and improved data acquisition efficiency.^[^ ^14^ ^]^ In this study, after establishing the MCAO‐induced RI mouse model, single‐nucleus RNA sequencing (snRNA‐seq) was employed to analyze transcriptional changes across various cell types and their subtypes. This approach allowed the identification of distinct cell populations and subpopulations in the retina following RI. Notably, the expression of high‐mobility group A2 (Hmga2) in Müller cells was significantly increased after RI. To further investigate the role of Hmga2 in RI, Müller cell‐specific *Hmga2* conditional knockout transgenic mice were generated. It was indicated that modulating Hmga2 expression in Müller cells significantly affected RGC apoptosis and neuroinflammation. Moreover, Müller glial cells were found to undergo autophagy after RI, and Hmga2 modulation influenced the extent of Müller cell autophagy via the phosphoinositide 3‐kinase (PI3K)/protein kinase B (AKT) pathway. Ultimately, *Hmga2* knockout promoted the expression of autophagy‐related proteins in Müller cells, thereby regulating neuroinflammation and mitigating RGC apoptosis.

Based on the aforementioned findings, we developed a nanomedicine by encapsulating Hmga2‐siRNA within Müller cell membrane‐coated liposomes, termed siRNA‐Hmga2@LMM. This formulation was administered via intravitreal injection following RI. The siRNA‐Hmga2@LMM significantly improved retinal function, representing a novel therapeutic strategy for patients experiencing unilateral painless vision loss.

## 2 Results

### 2.1 Identification of Cell Types in an MCAO‐Induced RI Mouse Model

**Figure 1:**
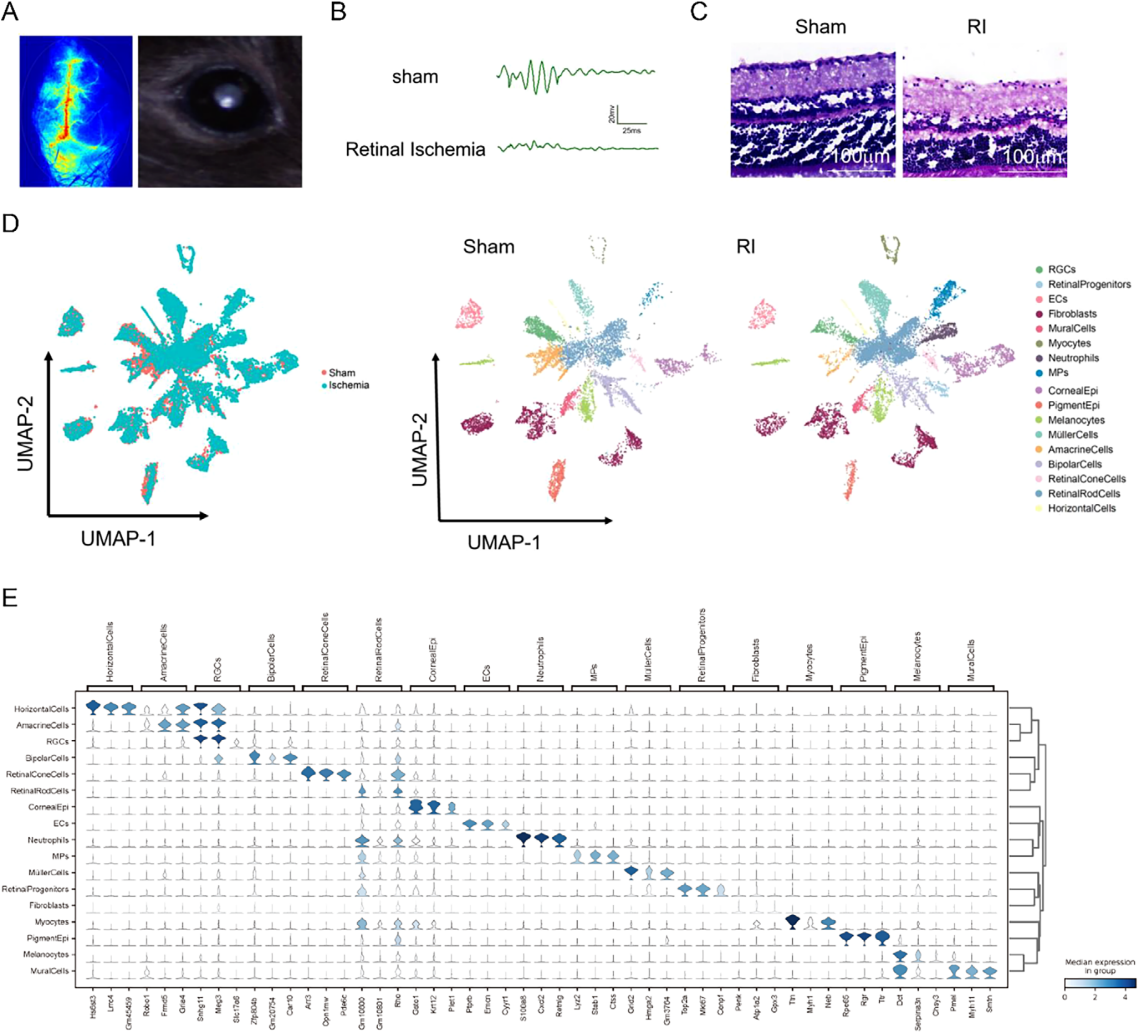
Single‐cell RNA sequencing of the retinal tissue of mice in the sham and RI groups. A) Representative photographs of Laser Speckle Contrast Imaging of right hemisphere ischemia induced by the MCAO model, accompany “Whitening” following MCAO model‐induced right RI. B) Representative images of right retinal OPs of mice in the sham and RI group. C) Representative images of HE staining on the right retina of mice in the sham and RI groups. D) UMAP plot showing the different cell types in the retinal tissue based on transcriptome data. Three mice per group were tested after mixing the samples. Each dot represents a cell. E) Violin plot showing cell type‐specific gene marker expression in the different cell clusters.

To establish a successful MCAO‐induced RI model, cerebral blood flow in the RI group was assessed using laser Doppler imaging. Induction of the model was evidenced by whitening of the right retina, as shown in **Figure** **1**A. Additionally, the oscillatory potentials (OPs) of the retina were measured in right‐whitened eyes. The amplitude of OPs in the RI group was significantly reduced or even absent compared to that in the sham group (Figure 1B), demonstrating successful generation of the MCAO‐induced RI model. Hematoxylin and eosin (H&E) staining revealed that, compared with that in the sham group, the total thickness of the retina was significantly reduced in the RI group at 24 h, accompanied by degeneration of RGCs (Figure 1C). Subsequently, samples were collected from the right eyes of mice in both the sham and RI groups for retinal tissue extraction.

To systematically analyze transcriptome differences between the sham and RI groups at the single‐cell level, right retinal samples from three mice per group were pooled for snRNA‐seq. After quality control, 9037 and 12401 cells were obtained from the retinas of mice in the sham and RI groups, respectively. Based on cell type‐specific markers, 18 distinct cell types were identified, including Müller cells, amacrine cells, bipolar cells, retinal cone cells, retinal rod cells, horizontal cells, RGCs, and retinal progenitors (Figure 1D,E).

### 2.2 Hmga2 Expression Is Upregulated in Müller Cells Following RI

**Figure 2:**
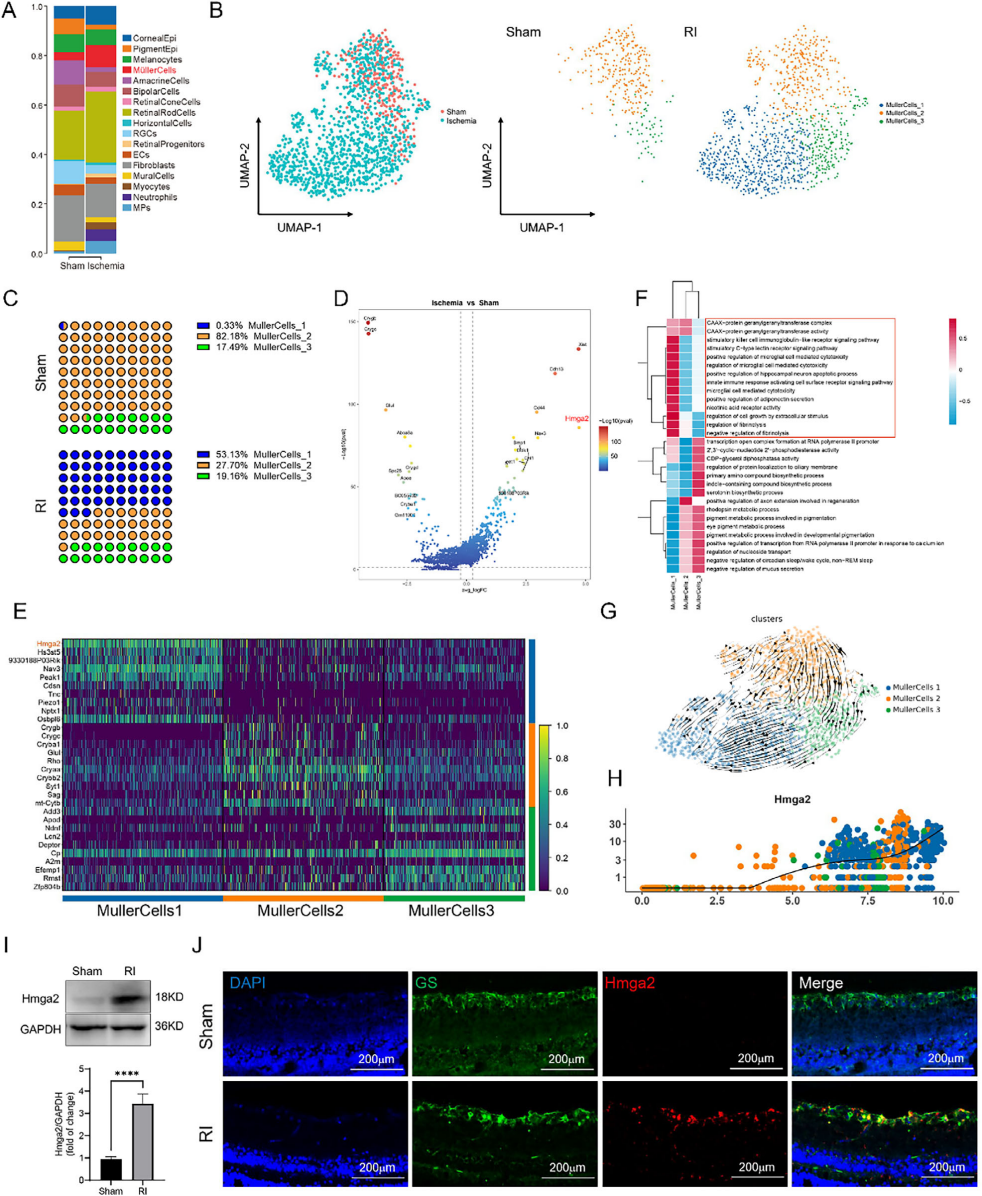
Validation that Hmga2 expression is upregulated in the Müller cells following RI. A) Bar graph showing the proportion of each cluster between two groups. B) UMAP plots for cell subset classification for Müller cells of each group. C) The proportion of different cell subsets of Müller cells between the two groups. D) Comparison of log p‐values of Müller cells differential expression of mice in the sham and RI group. The log p‐values sign corresponds to upregulation or downregulation. E) The ten most highly expressed genes among all Müller cell subpopulations. F) Heat map showing GSVA enrichment scores per cell of Müller cell subclusters. G) Pseudotime analysis (RNA velocity) of Müller cell subclusters in each group. H) Pseudotime analysis of the Hmga2 gene. I) Expression level of Hmga2 in Müller cells of retina 24 h after RI. J) Representative photographs of Hmga2 and GS co‐staining of frozen sections of the retina at 24 h after RI. For panel I: ^****^ *p* < 0.0001 by Student's *t* test. Data are expressed as the mean ± SD; n=6/group.

Müller cells, the predominant glial cells in the retina, play essential roles in modulating inflammatory responses and facilitating tissue repair after injury, making them critical for maintaining retinal health. As shown in **Figure** **2**A, the proportion of Müller cells in the ischemic retina significantly increased compared to that in the sham group (by 3.6‐fold). Therefore, a more detailed analysis of Müller cells was conducted. All Müller cells were reclustered into three cellular subpopulations based on their cell surface‐specific genes. Notably, a novel Müller cell subpopulation (subpopulation 1) emerged after RI, characterized by the highest expression of *Hmga2* (Figure 2B–E). Gene set variation analysis (GSVA) revealed that the primary biological functions of subpopulation 1 were closely related to inflammatory responses and cell death (Figure 2F). Furthermore, RNA velocity analysis within the pseudotime framework suggested that Müller cell subpopulation 2 may differentiate into subpopulation 1 following RI. Additionally, pseudotime gene expression analysis also indicated that *Hmga2* expression increased concurrently with the differentiation toward subpopulation 1 (Figure 2G,H). Finally, fluorescence‐activated cell sorting (FACS) of Müller cells revealed a significant upregulation of Hmga2 protein expression following RI (Figure 2I). Immunofluorescence (IF) analysis further confirmed a marked increase in Hmga2 protein levels in Müller cells after RI (Figure 2J). These findings suggest that upregulation of Hmga2 expression after RI may influence the inflammatory response of Müller cells, ultimately affecting retinal function.

### 2.3 *Hmga2* Knockout Improves Retinal Function Following RI

**Figure 3:**
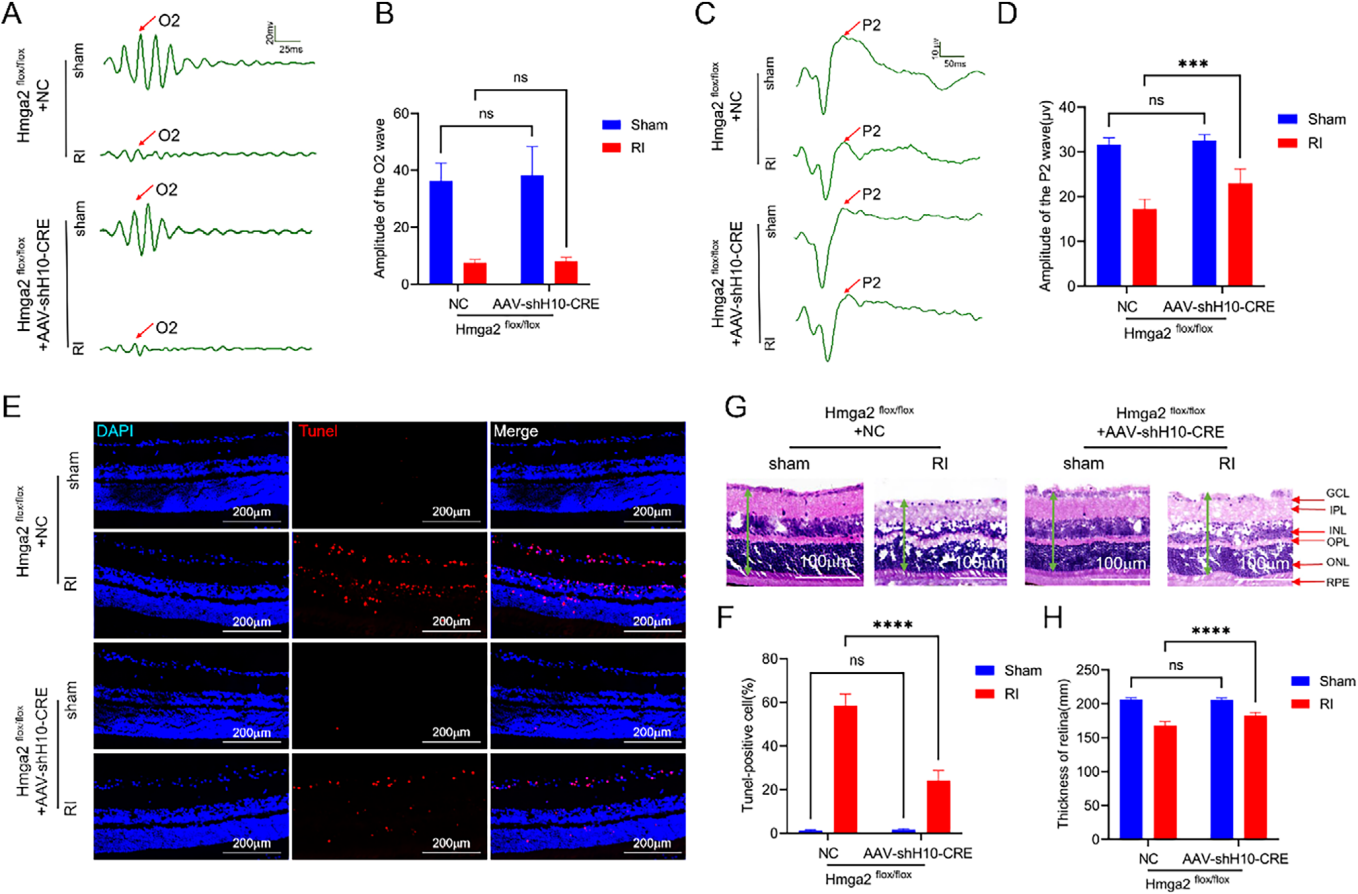
*Hmga2* knockout improves retinal function following RI. A) OPs of the right retina of each group. B) Quantification of the result in A. C) Representative FVEP of the right eye profile in each group. D) Quantification of the amplitude of the P2 wave in panel C. E) Representative photographs of apoptotic cells of frozen sections of the retina at 24 h after MCAO‐induced right RI. F) Quantification of result in panel E. G) Representative images of HE staining on the right retina of each group. H) Quantification of result in panel G. For panels B, D, F, H: ns, no significance, ^***^ p < 0.001, ^****^ p < 0.0001 by two‐way ANOVA analysis. Data are expressed as the mean ± SD; n = 6/group. GCL: ganglion cell layer; IPL: inner plexiform layer; INL: inner nuclear layer; OPL: outer plexiform layer; ONL: outer nuclear layer; RPE: retinal pigment epithelium.

To further validate the hypothesis regarding the regulatory role of Hmga2 following RI, mice with a Müller cell‐specific knockout of *Hmga2* were generated (Figure S1, Supporting Information) using an adeno‐associated virus (AAV) system. First, retinal OPs confirmed the successful establishment of the RI model in both knockout and non‐knockout mice, with no difference observed between the groups (**Figure** **3**A,B). Flash visual evoked potentials (FVEP) were measured 24 h after RI. The P2 wave amplitudes in the Hmga2^flox/flox^ + AAV‐shH10‐Cre group and Hmga2^flox/flox^ + NC group were 22.97 ± 3.24 and 17.20 ± 2.24 µV, respectively (Figure 3C,D). Terminal deoxynucleotidyl transferase dUTP Nick‐End labeling (TUNEL) staining confirmed that *Hmga2* knockout reduced apoptosis, particularly in RGCs, with TUNEL‐positive RGCs accounting for 24.17 ± 1.57% and 58.50 ± 5.43% in the *Hmga2* ^flox/flox^ + AAV‐shH10‐Cre and *Hmga2* ^flox/flox^ + NC groups, respectively (Figure 3E,F). Additionally, the thickness of the retina (marked by green arrows) following RI was 182.44 ± 4.80 and 167.76 ± 6.12 µm in the *Hmga2* ^flox/flox^ + AAV‐shH10‐Cre and *Hmga2* ^flox/flox^ + NC groups, respectively (Figure 3G,H).

**Figure 4:**
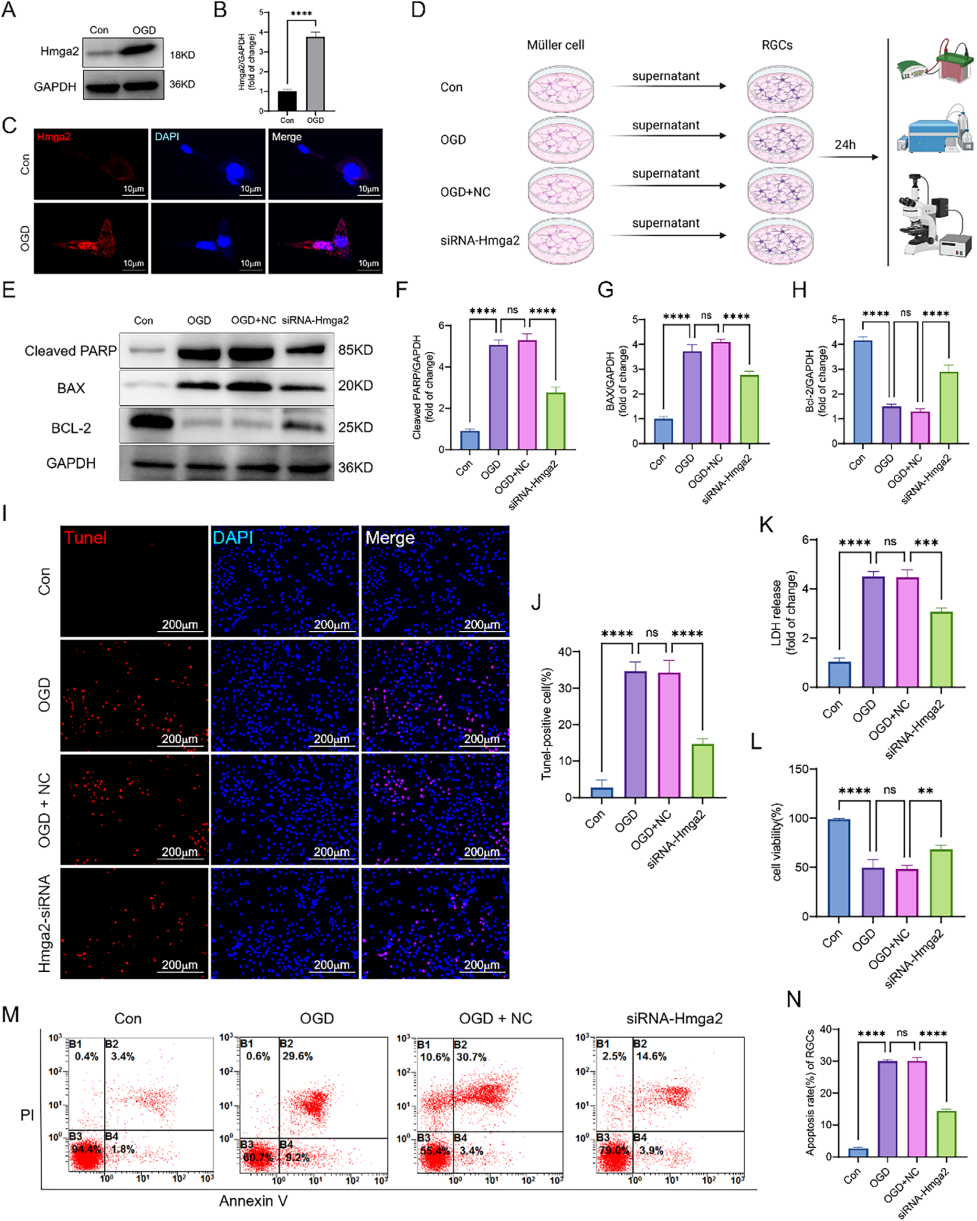
*Hmga2* knockdown in OGD‐induced Müller reduces RGCs death in vitro studies. A) Expression level of Hmga2 in Müller cell 6 h after OGD. B) Quantification of the result in A. C) Representative fluorescence images of Hmga2 localization in Müller cells after OGD. D) The in vitro protocol for detecting RGCs death regulated by knock down *Hmga2* in primary Müller cells. Primary Müller cells treated with NC or siRNA‐Hmga2 for 24 h. After successful transfection, the Müller cell culture medium was replaced with glucose‐free medium and further subjected to an OGD for 6 h. After removal of the supernatant, cells were cultured with complete culture medium for 24 h, and then the supernatant was collected as RGCs conditioned medium for 24 h. E) WB analysis of cleaved‐PARP, Bax, Bcl‐2 expression in RGCs in each group. F—H) Quantification of results in E. I, J) representative images I and quantification J of RGCs death based on Tunel assay in vitro of different groups. K) Cell viability test in different groups. L) LDH release detection in different groups. M) Apoptotic rate of RGCs in different groups by flow cytometry. N) Quantification of the result in M. For panel F‐H, J‐L, N: ns, no significance, ^**^ *p* < 0.01, ^***^ *p* < 0.001, ^****^ *p* < 0.0001 by one‐way ANOVA analysis. Data are presented as the mean ± SD, n = 3/group.

Essential functional cells such as RGCs are not only subjected to direct ischemic and hypoxic injury but also suffer secondary damage from inflammatory cytokines released by Müller cells into the extracellular environment following RI, thereby disrupting the microenvironment of RGCs and other retinal cells. To confirm the impact of Müller cell activation on RGCs after RI, the following in vitro experiments were conducted. First, as shown by the Cell Counting Kit‐8 (CCK‐8) assay, after 6 h of oxygen‐glucose deprivation (OGD) in Müller cells, cell viability decreased to ≈50%. Therefore, the OGD duration for subsequent in vitro experiments was set at 6 h (Figure S2, Supporting Information). Western blot (WB) and IF analyses confirmed that Hmga2 expression increased in Müller cells after 6 h of OGD in vitro (**Figure** **4**A–C). Next, conditioned medium (CM) from Müller cells transfected with Hmga2‐targeting siRNA (siRNA‐Hmga2) and subjected to OGD (successful knockdown confirmed via WB; Figure S3, Supporting Information) was then used to treat RGCs to evaluate the effects of *Hmga2* knockdown in Müller cells on RGCs (Figure 4D). WB analysis indicated a significant reduction in apoptosis‐related proteins in RGCs incubated with CM from *Hmga2*‐knocked‐down Müller cells (Figure 4E–H). TUNEL staining and flow cytometry analysis showed that, compared with that in the OGD alone group, RGC apoptosis was reduced when cultured with CM from Müller cells lacking *Hmga2* (Figure 4I,J,M,N). Furthermore, both cell viability and lactate dehydrogenase (LDH) levels in these RGCs were significantly improved compared to those incubated with CM from Müller cells after OGD without knockdown (Figure 4K,L). Collectively, these results demonstrate that *Hmga2* knockout in Müller cells reduces RGC death, mitigates cellular damage, and improves retinal function following RI.

### 2.4 *Hmga2* Knockout Ameliorates Neuroinflammation Following RI

**Figure 5:**
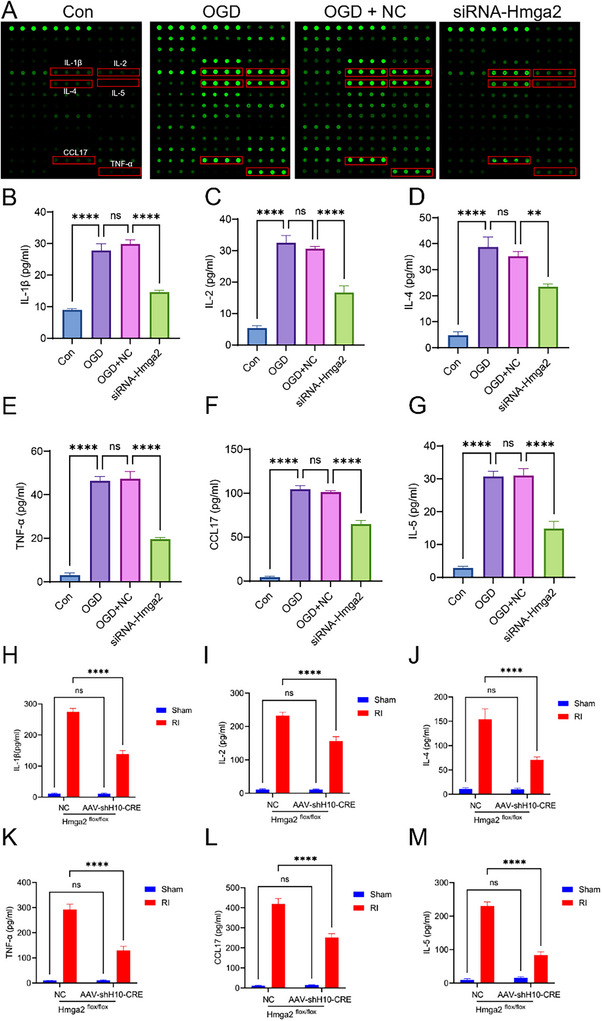
*Hmga2* knockout ameliorates neuroinflammation following RI. A) Representative experimental diagram of the chip slide carrier from CM of Müller cell in vitro in different groups. B–G) ELISA of IL‐1β, IL‐2, IL‐4, TNFα, CCL17, and IL‐5 in supernatants of Müller cells in vitro in different groups. H–M) ELISA of IL‐1β, IL‐2, IL‐4, TNFα, CCL17, and IL‐5 in retinal of mice in different groups. For panel B‐G: ^**^ *p* < 0.01, ^****^ *p* < 0.0001 by one‐way ANOVA analysis. Data are presented as the mean ± SD, n = 3/group. For H‐M: ns, no significance, ^****^ *p* < 0.0001 by two‐way ANOVA analysis. Data are presented as the mean ± SD; n =6/group.

Given the inflammation‐regulating role of Müller cells as glial components of the retina, we further analyzed the CM from Müller cells subjected to OGD under different treatment conditions using a high‐throughput mouse cytokine antibody array. The results revealed significant changes in inflammatory cytokines, including TNF‐α, IL‐1β, IL‐2, IL‐4, IL‐5, and CCL‐17 (**Figure** **5**A). To validate these findings, an enzyme‐linked immunosorbent assay (ELISA) was performed on CM from each treatment group. Compared to CM from Müller cells in the OGD group, the expression levels of these inflammatory cytokines were markedly reduced in CM from Müller cells after *Hmga2* knockdown (Figure 5B–G).

Additionally, these inflammatory cytokines were examined in vivo, revealing that, compared to that in the Hmga2^flox/flox^ + NC group, the expression of all six cytokines was significantly reduced in the Hmga2^flox/flox^ + AAV‐shH10‐Cre group (Figure 5H–M). These findings suggest that *Hmga2* knockout ameliorates neuroinflammation following RI.

### 2.5 *Hmga2* Knockout Promotes Autophagy via the PI3K/AKT Signaling Pathway in Müller Cells Following RI

**Figure 6:**
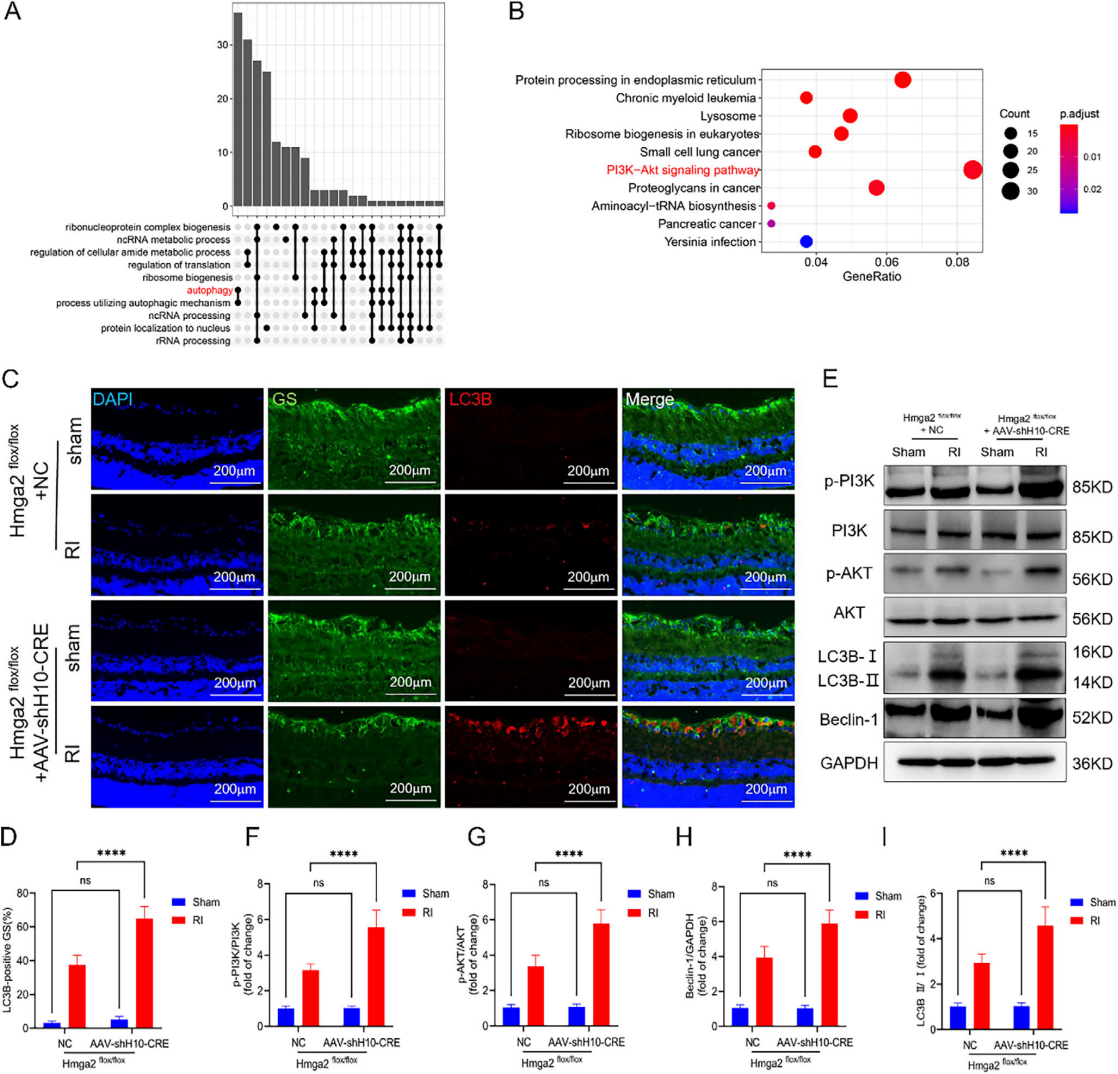
*Hmga2* knockout promotes autophagy in Müller cells following RI in vivo. A) The results of GO analysis of Hmga2^+^ Müller cell subset. B) The results of KEGG analysis of Hmga2^+^ Müller cell subset. C) GS/LC3B double immunostaining in the retinal tissue 24 h after RI. D) Quantification of results in C. E) WB analysis of p‐PI3K, p‐AKT, Beclin‐1, and LC3B II/I expression in retinal tissue in each group. F–I) Quantification of the result in E. For panel D, F‐I: ns, no significance, ^****^ *p* < 0.0001 by two‐way ANOVA analysis. Data are presented as the mean ± SD; n =6/group.

Single‐cell analysis of snRNA‐seq data revealed concurrent enrichment of autophagy‐related processes via Gene Ontology (GO) analysis and the PI3K/AKT signaling pathway via Kyoto Encyclopedia of Genes and Genomes (KEGG) analysis in Müller cell subpopulation 1 after RI (**Figure** **6**A,B). In vivo, IF staining of tissues showed that, compared with that in the Hmga2^flox/flox^ + NC group, the proportion of LC3B‐positive Müller cells increased significantly in the Hmga2^flox/flox^ + AAV‐shH10‐Cre group (Figure 6C,D). Moreover, WB analysis confirmed that, compared with that in the Hmga2^flox/flox^ + NC group, phosphorylation levels of PI3K/AKT pathway proteins were elevated, along with a significant increase in the LC3B‐II/I ratio and Beclin1 expression following *Hmga2* knockout (Figure 6E–I).

**Figure 7:**
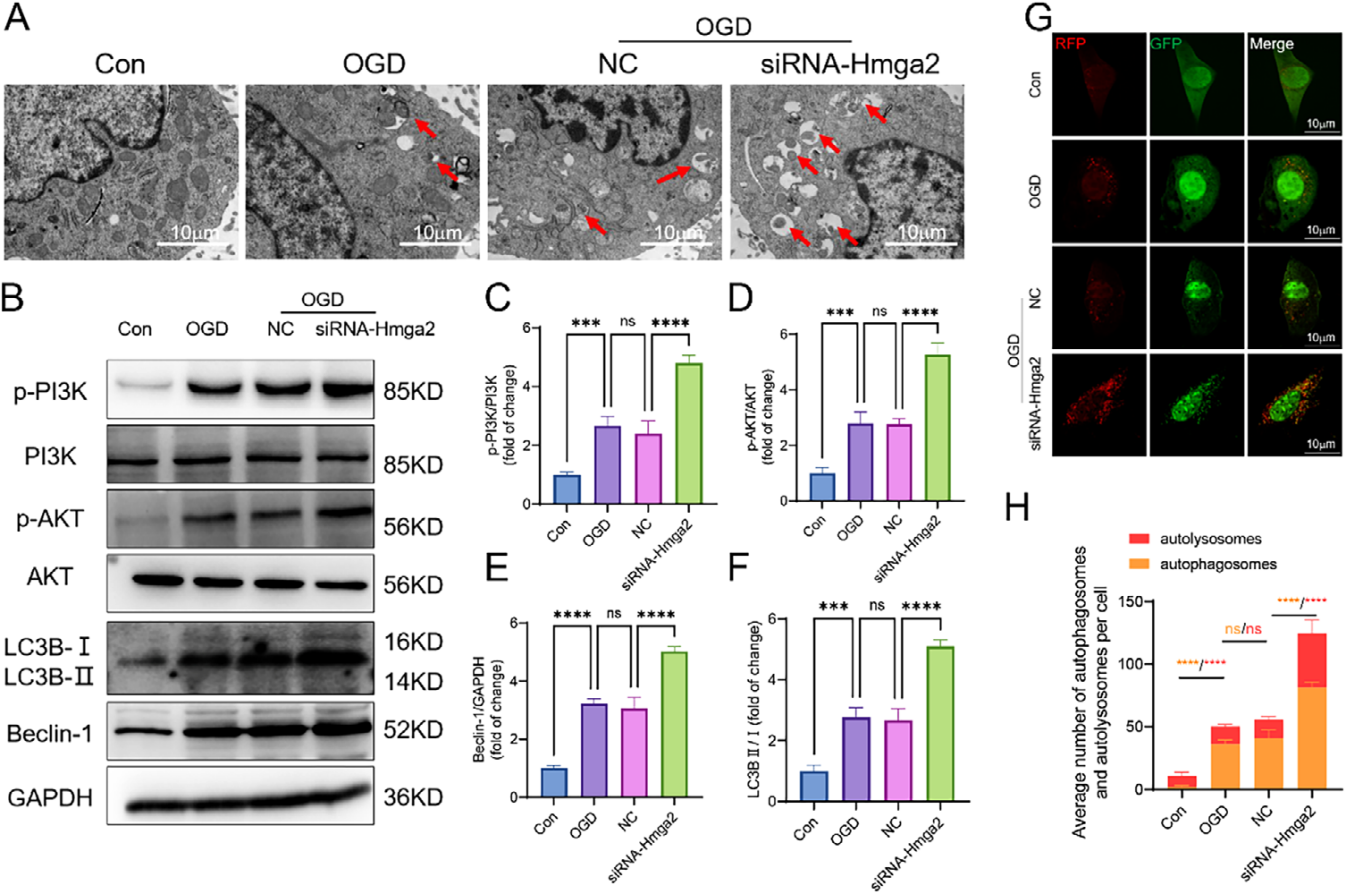
*Hmga2* knockdown enhances autophagy in Müller cells at 6 h after OGD in vitro. A) TEM photomicrograph of the Müller cell in each group. B) WB analysis of p‐PI3K, p‐AKT, Beclin‐1, and LC3B II/I expression in Müller cells in each group. C–F) Quantification of result in B. G) The autophagy levels of Müller cells detected with mRFP‐GFP‐LC3 after OGD. H) Quantification of the numbers of autophagosomes (yellow puncta) and autolysosomes (red puncta) per cell. For panel C‐H: ns, no significance, ^***^ *p* < 0.001, ^****^ *p* < 0.0001 by one‐way ANOVA analysis. Data are presented as the mean ± SD; n =3/group.

Concurrently, in vitro transmission electron microscopy (TEM) showed enhanced autophagy in Müller cells following *Hmga2* knockdown compared to those subjected to OGD alone (**Figure** **7**A). WB analysis indicated that the expression of autophagy pathway proteins associated with PI3K/AKT signaling was significantly increased in Müller cells after *Hmga2* knockdown compared with those subjected to OGD alone (Figure 7B–F). To investigate changes in autophagic flux, Müller cells were transduced with an adenovirus expressing mRFP‐GFP‐LC3. The number of both autophagosomes (yellow puncta) and autolysosomes (red puncta) increased after OGD, and *Hmga2* knockdown further enhanced the formation of both (Figure 7G,H).

**Figure 8:**
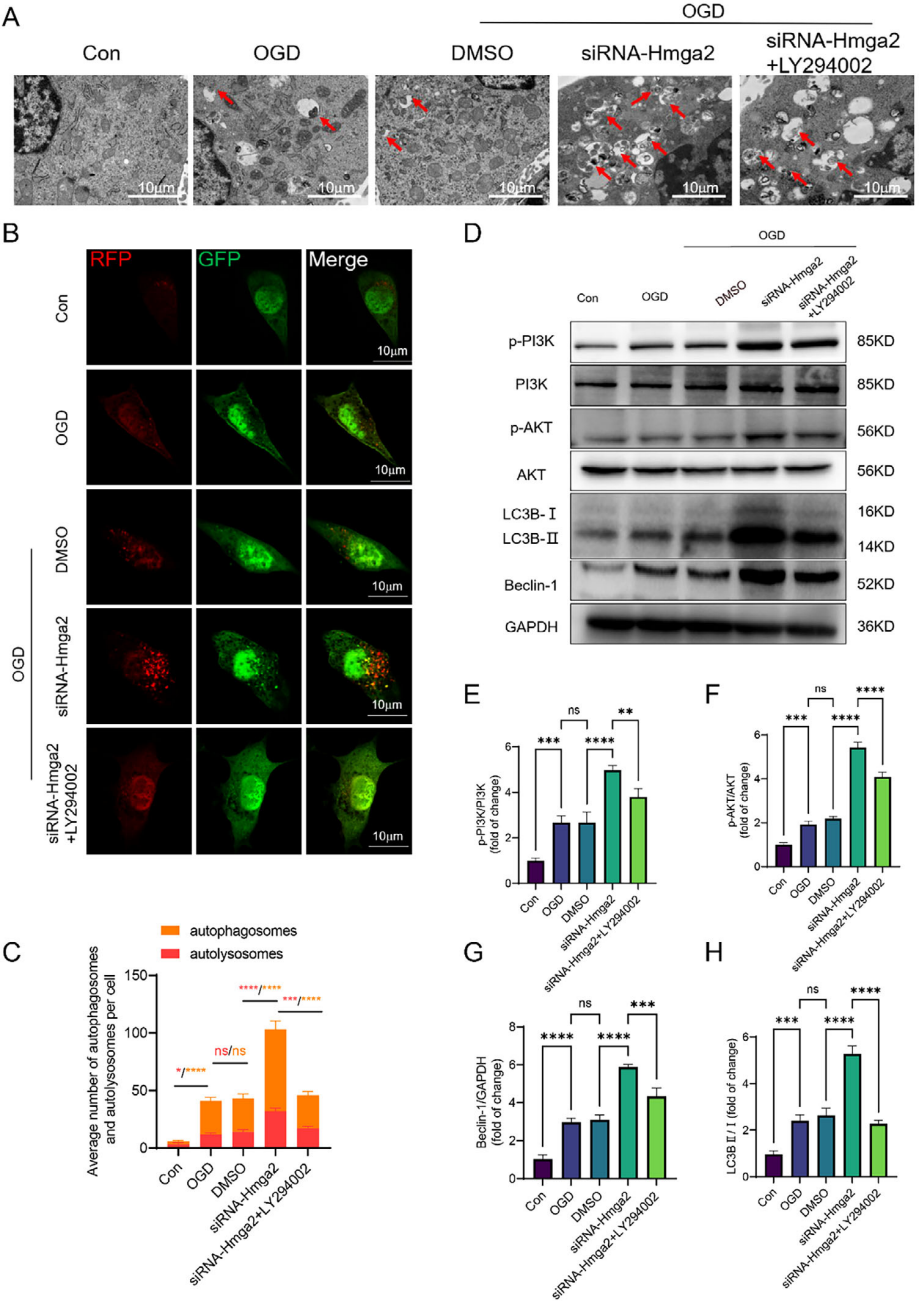
Modulation of PI3K/AKT signaling regulates OGD‐induced autophagy activity in Müller cells in vitro. A) TEM photomicrograph of the Müller cell in each group. B) The autophagy levels of Müller cells were detected with mRFP‐GFP‐LC3 after OGD. C) Quantification of the numbers of autophagosomes (yellow puncta) and autolysosomes (red puncta) per cell. D) WB analysis of p‐PI3K, p‐AKT, Beclin‐1, and LC3B II/I expression in Müller cells in each group. E–H) Quantification of results in D. For panel C, E‐H: ns, no significance, ^*^ *p* < 0.05, ^**^ *p* < 0.01, ^***^ *p* < 0.001, ^****^ *p* < 0.0001 by one‐way ANOVA analysis. Data are presented as the mean ± SD; n = 3/group.

To validate PI3K/AKT‐mediated autophagy in Müller cells after OGD, LY294002 (a PI3K inhibitor) was used in vitro. TEM, WB, and adenovirus‐mediated autophagy dual labeling demonstrated that PI3K inhibition reduced the expression of downstream autophagy‐related proteins, ultimately affecting autophagy in Müller cells (**Figure** **8**A–H).

### 2.6 Hmga2 May Regulate PI3K Phosphorylation Through Direct Binding

**Figure 9:**
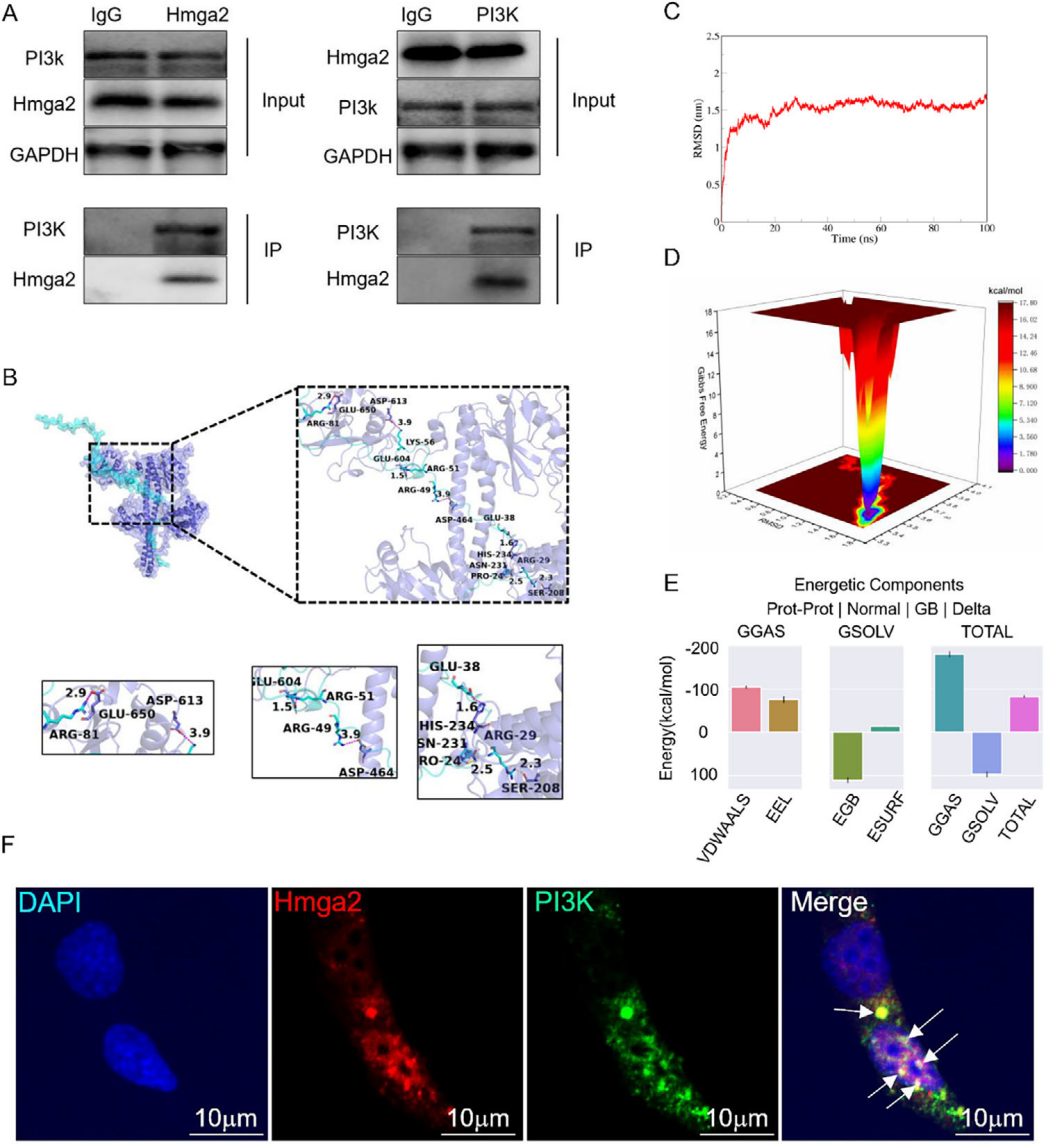
The Hmga2 protein binds PI3K. A) The results of co‐IP analysis confirming the interaction between Hmga2 and PI3K. B) Top: The PI3K protein is represented as a slate cartoon model, Hmga2 protein is shown as a cyan cartoon model, and their binding sites are shown as the corresponding‐colored stick structure. When focusing on the binding region, the binding site is then shown as a color of the protein to which it belongs. Down: magnified view of the boxed area. C) RMSD curve of Hmga2/PI3K complex. D) Gibbs Free Energy Landscape of Hmga2/PI3K complex. E) The average binding free energy of Hmga2/PI3K complex. VDWAALS: van der Waals Energy, EEL: Electrostatic Energy, EGB: Polar Solvation Energy (GB Model), ESURF: Nonpolar Solvation Energy, GGAS: Gas‐Phase Free Energy, GSOLV: Solvation Free Energy, TOTAL: Binding Free Energy. F) IF micrographs showing the co‐localization of Hmga2 and PI3K in the Müller cells.

To investigate whether Hmga2 modulates PI3K phosphorylation via direct binding, co‐immunoprecipitation (co‐IP) was first performed in Müller cells, demonstrating a physical interaction between Hmga2 and PI3K (**Figure** **9**A). Subsequently, protein–protein interaction analysis using PyMOL identified and classified all functional residues based on interaction type. Among the hydrogen bond interactions, multiple residue pairs were observed to form hydrogen bonds between PI3K and Hmga2, such as ASN231 of PI3K and PRO24 of Hmga2. These interactions resulted in a PI3K‐Hmga2 binding score of −485, indicating strong interaction strength (Figure 9B). In addition, molecular dynamics simulations were performed to analyze the root‐mean‐square deviation (RMSD) trajectory of the complex over a 100 ns simulation period. The RMSD gradually increased to ≈1.5 nm within the first 20 ns, fluctuated within a narrow range, and then stabilized, indicating that the system quickly reached equilibrium after initial conformational relaxation and maintained structural stability without significant perturbations (Figure 9C,D). The average total binding free energy of the Hmga2−PI3K complex was −82.46 kcal mol^−1^, suggesting strong binding affinity and a thermodynamically favorable interaction (Figure 9E). Also, biolayer interferometry assays showed that Hmga2 binds to PI3K with a Kd of 12 nM (Figure S4, Supporting Information). These results confirmed the system's favorable simulation and binding stability. Furthermore, confocal microscopy provided additional evidence supporting direct binding between Hmga2 and PI3K (Figure 9F). Collectively, these findings suggest that following RI, *Hmga2* knockout promotes PI3K phosphorylation, thereby regulating autophagy in Müller cells, modulating neuroinflammation, reducing RGC death, and ultimately mitigating RI injury.

### 2.7 siRNA‐Hmga2@LMM Confers Neuroprotection and Supports Functional Recovery Following RI

**Figure 10:**
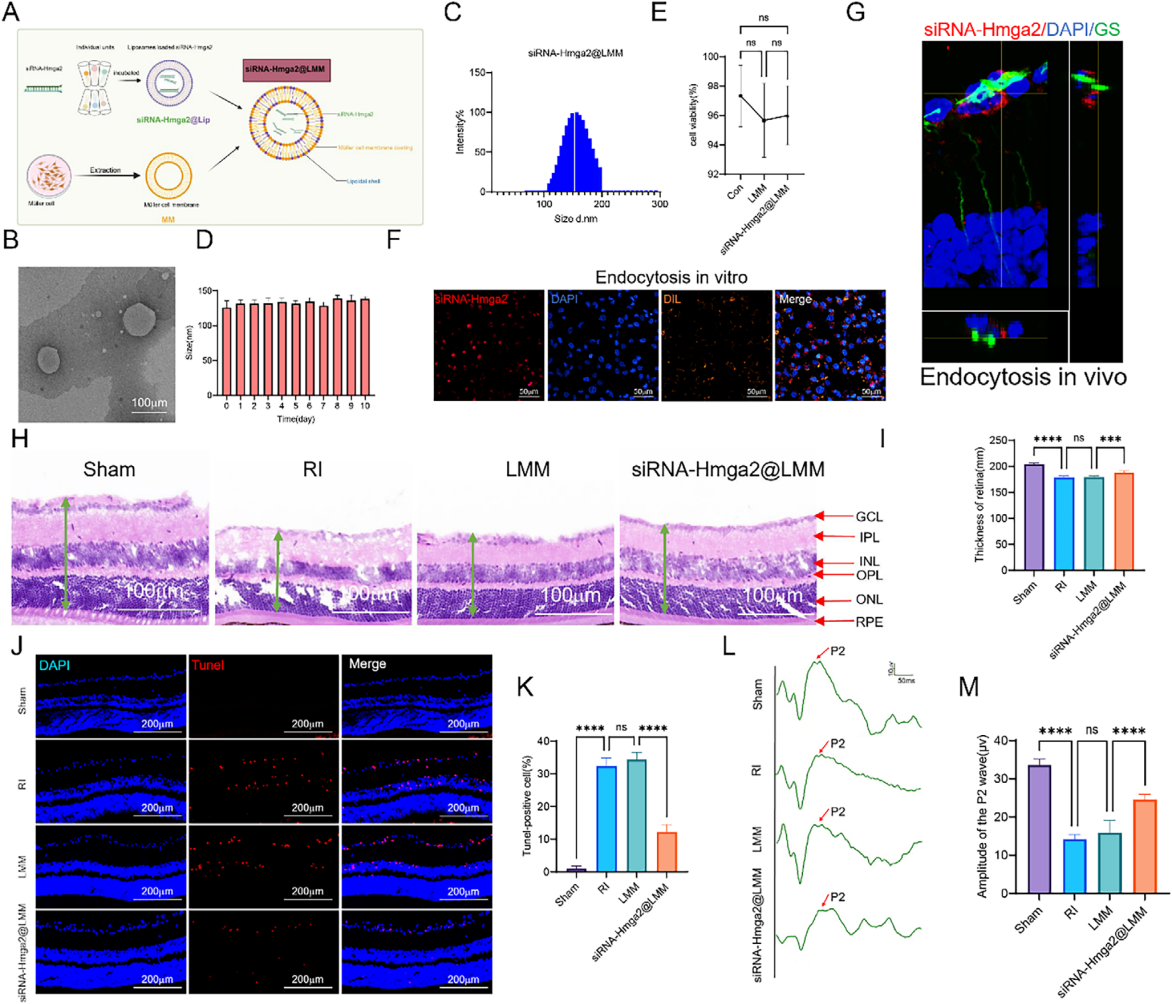
Nanomaterials carrying siRNA‐Hmga2 confer neuroprotection and functional recovery following RI. A). Schematic diagram representing the generation of si‐Hmga2@LMM Nanomaterials. B) TEM images of the si‐Hmga2@LMM. C) The diameter distribution of si‐Hmga2@LMM nanomaterials. D) Stability test of si‐Hmga2@LMM Nanomaterials. E) Biological toxicity test of si‐Hmga2@LMM nanomaterials in vitro. F) The uptake of nanomaterials by Müller cells in vitro. G) The uptake of nanomaterials by Müller cells in vivo. H) Representative images of HE staining on the right retina of the effects of different treatments. I) Quantification of results in H. J) Representative photographs of apoptosis cells of the retina of different treatments. K) Quantification of result in J. L) Representative FVEP of right eye profile in different groups. M) Quantification of the result in L. For panel E, I, K, M: ns, no significance, ^***^ *p* < 0.001, ^****^ *p* < 0.0001 by one‐way ANOVA analysis. Data are presented as the mean ± SD; n = 6/group.

Although various therapeutic strategies are available for CRAO, consistently effective treatments remain limited. siRNAs are a class of RNA‐based therapeutics capable of inhibiting gene expression to improve or alleviate disease symptoms.^[^ ^15^ ^]^ They are negatively charged macromolecules that cannot easily bind to the cell surface or penetrate the cell membrane.^[^ ^16^ ^]^ To address these limitations, siRNAs have been encapsulated using a variety of materials such as lipids, polymers, inorganic substances, proteins, and their combinations to enhance their delivery efficiency.^[^ ^17^ ^]^ The fusion of synthetic nanocarriers with natural cell membranes can mimic the surface characteristics and functions of source cells, further enhancing their targeting accuracy and therapeutic efficacy.^[^ ^18^ ^]^ Based on these considerations, we developed a nanotechnology‐based strategy (**Figure** **10**A). First, lipid nanoparticles were used as nanocarriers to load negatively charged siRNA‐Hmga2 (siRNA‐Hmga2@Lip). Next, Müller cell membranes (MM)were extracted and fused with the lipid nanocarrier membrane to form a hybrid membrane, which was further used to coat the siRNA‐Hmga2@Lip, producing siRNA‐Hmga2@LMM. The final formulation achieved a loading efficiency of 4.8% and an encapsulation efficiency of 83.12%.

An electron microscopy image of the siRNA‐Hmga2@LMM nanomedicine is shown in Figure 10B. The particle size of siRNA‐Hmga2@LMM was 157.07 ± 10.62 nm (Figure 10C, Figure S5, Supporting Information), with a PDI of 0.20 ± 0.02 and a zeta (ζ) potential of 26.39 ± 4.09 mV. Stability testing indicated that the formulation remained stable for at least 10 d (Figure 10D), demonstrating good physicochemical stability. In addition, cumulative release of siRNA‐Hmga2@LMM is shown in Figure S6 (Supporting Information). In vitro experiments showed that siRNA‐Hmga2@LMM exhibited no cytotoxicity (Figure 10E). Both in vivo and in vitro studies demonstrated that siRNA‐Hmga2@LMM effectively delivered siRNA‐Hmga2 into Müller cells (Figure 10F,G).

To evaluate the therapeutic efficacy of the nanomedicine, mice were randomly divided into four groups: sham, RI, LMM (empty load group), and siRNA‐Hmga2@LMM. 5 min after RI induction, the nanomedicine (2 µL, 20 µg µL^−1^) was administered via intravitreal injection, and retinal samples were collected 24 h post‐ischemia for analysis. In vivo, retinal thicknesses (marked with green arrows) in the sham, RI, LMM, and siRNA‐Hmga2@LMM groups were 204.1 ± 2.44, 178.4 ± 2.94, 179.6 ± 2.39, and 187.8 ± 3.75 µm, respectively (Figure 10H,I). The proportions of TUNEL‐positive cells were 1.00 ± 0.89, 32.33 ± 2.58, 34.33 ± 2.25, and 12.17 ± 2.32% across the same groups (Figure 10J,K). The P2 amplitudes were 33.60 ± 1.5, 14.2 ± 1.64, 15.88 ± 3.31, and 24.58 ± 1.40 µV across these groups (Figure 10L,M). In summary, these results indicate that siRNA‐Hmga2@LMM confers neuroprotection and supports functional recovery in mice following RI.

## 3 Discussion

The sudden loss of vision in one eye due to CRAO, commonly referred to as an eye stroke, causes profound psychological distress in patients and imposes substantial economic burdens on society. However, the underlying molecular mechanisms of this condition remain largely uncharacterized, and RI is thought to be a primary contributing factor.

Currently, to study RI, various animal models with distinct characteristics are carefully selected and employed. For instance, models involving clamping or ligating the CRA carry the risk of damaging the optic nerve, which may exacerbate symptoms of ocular ischemia and confound investigations specifically targeting the ischemic retina. Additionally, the mouse model using elevated intraocular pressure is commonly used to study ischemic damage in acute primary angle‐closure glaucoma. However, retinal damage caused by mechanical compression from saline injection may introduce confounding variables. Furthermore, the thermal effects from laser‐induced RI can directly damage retinal tissue, thereby compromising the interpretation of ischemic pathology in isolation. Additionally, a major limitation of photochemically induced RI is the systemic phototoxicity of photosensitizers in mice, along with the hepatorenal toxicity associated with their administration via tail vein injection. Notably, both models require specialized equipment and substantial technical expertise. In our study, we employed the MCAO‐induced RI model, which closely mimics the acute, painless monocular vision loss associated with ICA occlusion observed in clinical settings. This approach is relatively straightforward and induces RI through the interruption of blood flow, without inflicting mechanical damage to the retina or optic nerve. Importantly, this model provides a valuable alternative perspective for investigating RI pathophysiology.

In recent years, Hmga2 has emerged as a key focus in diverse fields such as cancer biology, developmental biology, and gene regulation. Hmga2 has been shown to play a critical role in tumor progression by accelerating cell proliferation, inhibiting apoptosis, and enhancing cell migration and invasion.^[^ ^19^ ^]^ Specifically, Hmga2 facilitates colorectal cancer progression by regulating STAT3‐mediated recruitment of tumor‐associated macrophages.^[^ ^20^ ^]^ Furthermore, downregulation of Hmga2 expression in ischemic brain injury has been reported to reduce infarct size, inflammatory responses, and neuronal apoptosis in mice, thereby exerting neuroprotective effects.^[^ ^21^ ^]^ Another study indicates that Hmga2 knockdown regulates the level of autophagy to inhibit malignant peripheral nerve sheath tumors growth.^[^ ^22^ ^]^ To the best of our knowledge, we identified for the first time—via high‐throughput snRNA‐seq—that Hmga2 is significantly upregulated following RI. Subsequent in‐depth experiments confirmed that Hmga2 knockout alleviates RI. Particularly, knockout of Hmga2 attenuated inflammation in Müller cells, thereby reducing RGC death (58.6% reduction in mortality, p < 0.0001) and mitigating retinal thinning (8% attenuation of thickness loss, p < 0.0001). Collectively, these effects improved retinal function following RI. Similarly, genetic ablation of Hmga2 alleviated RI‐induced retinal pathology, consistent with previous reports on Hmga2's role in cerebral ischemic injury. Taken together, these findings highlight Hmga2 as a pathogenic driver of ischemic tissue damage. We further detected transcription factors that may bind to the Hmga2 promoter and regulate the expression of Hmga2 using DNA pull‐down MS. Database analysis identified six likely candidates: Max, Patz1, Sp3, Maz, Hic2, and Zkscan3. Among them, the Max transcription factor exhibited the highest fold change; therefore, we validated Max's regulatory effect on Hmga2 (Figure S7, Supporting Information). We demonstrated that Max may act upstream of Hmga2, providing additional insight into Hmga2 regulation in the RI model. However, the roles of the remaining five transcription factors should be investigated in future studies to better understand the involvement of Hmga2 following RI. Moreover, other upregulated genes aside from Hmga2 also drew our attention. In addition to presenting the top 10 highly expressed genes in the Müller1 subcluster (Figure 2E), we comprehensively analyzed multiple Müller cell‐enriched genes primarily involved in regulating inflammatory responses, cell cycle progression, and glutamatergic signaling. However, a literature review revealed that few studies have addressed the roles of these upregulated genes in the retinal ischemic response. Besser et al. indicated that Tnc is upregulated early in glial cells following RI, and its knockout alleviated retinal functional impairment.^[^ ^23^ ^]^ Another study showed that in a model of retinal degeneration, CD44 expression in Müller cells increases, and CD44 knockout exacerbates photoreceptor degeneration, retinal dysfunction, and inflammatory responses.^[^ ^24^ ^]^ Notably, interactions between these upregulated molecules and Hmga2 following RI have not been previously reported. In our study, we demonstrated for the first time a role for Hmga2 in Müller cells following RI. Future investigations should aim to determine direct promoter binding by Hmga2 using chromatin immunoprecipitation sequencing, explore indirect regulatory pathways using combinatorial genetic manipulation assays, and elucidate feedback mechanisms through protein interaction studies. These approaches may establish a hierarchical framework for the regulatory logic of gene networks in Müller cells in response to ischemia. Finally, although Xist shows a similar FC value with Hmga2 but a more significant p‐value in Figure 2D, its fold change was lower than that of Hmga2 (avg_logFC = 4.73 VS 4.76). Subsequently, during subclustering analysis of Müller cells, Xist was excluded from the top ten marker genes defining Müller subpopulation 1. Most critically, Xist functions as a long non‐coding RNA (lncRNA) essential for X‐chromosome silencing. Given this inherent biological role, Xist is fundamentally unsuitable as a therapeutic intervention target.

Furthermore, molecular dynamics simulations, co‐IP, and IF results revealed that Hmga2 may directly bind to PI3K. Therefore, Hmga2 likely occupies the phosphorylation site(s) of PI3K. Upon *Hmga2* knockout, these phosphorylation sites may become exposed, thereby initiating the downstream autophagy pathway. This activation promotes autophagy in Müller cells, modulates inflammatory responses, and ultimately mitigates RGC apoptosis while improving retinal function. Although molecular docking supported the Hmga2–PI3K interaction—with a Gibbs free energy change (ΔG = −82.46 kcal mol^−1^)—thermodynamic profiling remained incomplete due to experimental limitations. Whether Hmga2 specifically binds to the phosphorylation site(s) of PI3K requires confirmation in future investigations.

In our in vitro study, we observed for the first time that Müller cells primarily secreted six inflammation‐related cytokines after OGD, namely TNF‐α, IL‐1β, IL‐2, IL‐4, IL‐5, and CCL‐17. TNF‐α and IL‐1β, as classical pro‐inflammatory cytokines involved in ischemic injury, have been extensively reported as biomarkers for diagnosis and treatment. Their elevated concentrations in the culture supernatant of Müller cells were also confirmed in our experiments, indicating a pro‐inflammatory response following cellular damage. IL‐2 exhibits immunoregulatory functions, and in post‐cerebral ischemia, an IL‐2 monoclonal antibody can inhibit the infiltration of peripherally derived CD8^+^ T cells and improve long‐term sensorimotor function.^[^ ^25^ ^]^ Previous research has indicated that in RI‐related conditions, IL‐4 can enhance the survival of RGCs after ischemia by promoting the anti‐inflammatory phenotype of M2 microglia.^[^ ^26^ ^]^ Another study has shown that IL‐5 promotes eosinophil accumulation, thereby facilitating recovery of cardiac dysfunction after myocardial infarction.^[^ ^27^ ^]^ A recent study has suggested that CCL‐17 may serve as an inflammatory marker of ischemic brain injury severity, with its concentration decreasing as the injury improves.^[^ ^28^ ^]^ Based on these changes in inflammatory cytokine expression, we hypothesized that OGD‐induced damage to Müller cells enhances their pro‐inflammatory signaling, leading to increased secretion of pro‐inflammatory cytokines. Concurrently, this response also promotes the secretion of anti‐inflammatory cytokine IL‐4, along with changes in the expression of immunoregulatory cytokines IL‐2 and IL‐5. This cascade ultimately maintains homeostasis in the extracellular environment post‐injury. When Hmga2 expression is downregulated in vitro, the inflammatory response is correspondingly reduced, as evidenced by the decreased levels of inflammatory cytokines. This further supports the single‐cell analysis, which indicates that the Hmga2‐positive Müller cell subset is primarily associated with cell apoptosis and the infiltration of inflammatory cells.

Currently, the integration of synthetic nanocarriers with natural cell membranes has been applied in various disease models, including cancer and inflammation.^[^ ^29^ ^]^ Hybridization of liposomes with cell membranes can further enhance the biocompatibility of these materials.^[^ ^30^ ^]^ The cell membrane surface contains specific receptors, glycoproteins, and other proteins that facilitate the recognition of target cells by liposomes, thereby enabling targeted delivery.^[^ ^31^ ^]^ Upon hybridization with cell membranes, the resulting liposomes more effectively protect siRNA from degradation in vivo^[^ ^32^ ^]^ and enhance the ability of drugs to penetrate cell membranes.^[^ ^33^ ^]^ This allows siRNA to reach target cells more efficiently and exert gene‐silencing effects. Moreover, liposomes can reduce the metabolic degradation of drugs in the body, thereby prolonging their duration of action and reducing the frequency of administration.^[^ ^34^ ^]^ However, liposomes are easily degraded and have limited ability to accumulate in the body long‐term, which helps ensure treatment safety. Based on our previous investigation of membrane fusion between cell membranes and liposomes, we fabricated hybrid membrane nanocarriers formed by the fusion of Müller cell membranes and liposomes in this study. These hybrid nanocarriers encapsulated negatively charged siRNA‐Hmga2. In vitro experiments confirmed that siRNA‐Hmga2 was effectively endocytosed by Müller cells without affecting cell viability. Additionally, the drug delivery method used in this study was intravitreal injection, which allows direct delivery of drugs into the eye—particularly advantageous for treating diseases of the retina and vitreous. This localized administration bypasses the liver, avoiding systemic side effects, and enables the drug to reach high concentrations at the target site, thereby enhancing its therapeutic efficacy. Importantly, sustained drug release within the vitreous cavity prolongs therapeutic effects and reduces the need for frequent dosing.

In the treatment of RI, numerous methods in nanoparticles have been explored to date. For instance, one study demonstrated that nanoparticles derived from *Lycium barbarum* polysaccharides can inhibit RGC ferroptosis and microglial activation, thereby preserving visual function in RI models.^[^ ^35^ ^]^ Another study confirmed that supramolecular deferoxamine Crisbalor nanoparticles target ferroptosis, inflammation, and oxidative stress in the treatment of RI.^[^ ^36^ ^]^ In contrast, our siRNA‐Hmga2@LMM was proposed and applied for the treatment of RI via intravitreal injection—a highly innovative drug delivery strategy. Its core advantage lies in the integration of bio‐membrane and synthetic material properties to achieve efficient and precise retinal‐targeted therapy. Furthermore, this approach is not only applicable to RI but is also useful for treating fundus diseases such as DR and age‐related macular degeneration, offering new insights and potential therapeutic options for ocular diseases. Ultimately, this method provides a new mode of precise drug delivery and disease‐specific drug targeting. However, despite significant progress, several challenges remain. For example, ultracentrifugation (> 100 000 × *g*) during cell membrane extraction can lead to the shedding of transmembrane proteins, thereby compromising targeting efficiency. Second, the challenge of cross‐species dose translation arises, most notably due to significant differences in retinal thickness between mice and humans, which leads to species‐specific differences in vector penetration efficiency. Moreover, the safety of nanomaterial‐based drugs in humans remains largely unexplored and requires further investigation. The structural integrity and functional stability of LMM nanoparticles within the dynamic and complex human physiological environment over clinically relevant durations should be ensured. Uncontrolled degradation or premature siRNA release could compromise therapeutic efficacy or cause off‐target effects. In addition, precise control over biodistribution is essential to maximize retinal delivery while minimizing accumulation in non‐target organs. To design next‐generation nanoparticles with enhanced stability, biocompatibility, and biodegradability, refining the LMM platform and exploring novel hybrid or composite materials will be crucial. Our innovative siRNA‐Hmga2@LMM construct marks a significant advancement in retinal nanomedicine. Although current limitations and barriers to clinical translation remain, ongoing progress in nanomaterial design, delivery strategies, and safety evaluation methods offers strong potential for overcoming these challenges.

Although this study demonstrates that Hmga2 can regulate Müller cell autophagy via the PI3K/AKT signaling pathway, modulate neuroinflammation, and ultimately protect retinal function in the MCAO‐induced RI model, certain limitations must be acknowledged. First, our investigation focused exclusively on the intrinsic changes in Müller cells within the injured environment and their impact on RGCs, without examining alterations in other retinal cell types such as rod and cone cells. Differentially expressed genes (DEGs) in these other cell types should be further explored and may serve as potential targets for future research to broaden our understanding of the retinal cellular landscape following RI. Additionally, although Hmga2‐positive Müller cells were identified through snRNA‐seq, the mechanistic role of this subpopulation in RI was not examined at the cellular level owing to the limitations of the experimental platform. Furthermore, this study focused solely on the role of Müller cells as inflammatory regulators in secondary inflammatory damage following RI, without exploring the direct interaction mechanism between Müller cells and RGCs—an area that may offer valuable insights in future research. Moreover, co‐IP and molecular docking analyses revealed a physical interaction between Hmga2 and PI3K, and potential binding sites were computationally predicted. However, further investigation is required to determine whether Hmga2 competitively binds to the phosphorylation site(s) of PI3K, thereby influencing the downstream autophagy pathways. Finally, a limitation of our study is the inability to perform dynamic, real‐time imaging of Müller cell uptake of nanomaterials in vivo, due to equipment constraints and technical challenges.

In our study, the presence of non‐resident cells in single‐cell retinal suspensions was most likely due to inclusion during tissue sampling. To preserve the integrity of retinal tissue and avoid losing critical cells—particularly from the peripheral area—we slightly extended our sampling beyond strict retinal boundaries, which may have introduced trace amounts of adjacent choroidal tissue. Nevertheless, the primary objective of this study was to elucidate the role of Hmga2 in retinal Müller cells. The experimental design, including functional validation (such as gene knockout and cell‐specific analyses), and all conclusions were focused exclusively on retinal Müller cells. Throughout all subsequent analyses (such as differential expression and functional enrichment analyses), we consistently concentrated on cell types intrinsic to the retina, with particular emphasis on Müller cells.

In summary, our study demonstrates that following MCAO‐induced RI, Hmga2‐positive Müller cells emerge as a key subpopulation mediating cell death and inflammatory responses. Mechanistically, Hmga2 upregulation is tightly controlled by the transcription factor Max. Deletion of *Hmga2* reduces apoptosis in RGCs and attenuates neuroinflammatory reactions. Further analyses suggest that Müller cells undergo autophagy, which is enhanced via the PI3K/AKT signaling pathway upon Hmga2 knockout. Notably, Hmga2 may regulate downstream autophagy‐related pathways by directly interacting with PI3K. Hmga2 knockout was confirmed to enhance autophagic activity in Müller cells, ameliorate neuroinflammation, and decrease RGC apoptosis, thereby promoting functional recovery following RI. Notably, we developed a novel nanoparticle strategy by fusing Müller cell membranes with liposomes to deliver siRNA‐Hmga2, aiming to mitigate retinal dysfunction post‐RI. Our findings lay the groundwork for innovative therapeutic strategies for the treatment of RI and related retinal diseases.

## 4 Experimental Section

### Ethics Approval and Consent to Participate

All experimental procedures were approved by the Ethics Committee and Institutional Review Board of the Air Force Medical University (approval No. IACUC‐20220630). Animal experiments were conducted in accordance with the *Guide for the Care and Use of Laboratory Animals* published by the National Institutes of Health (NIH; Bethesda, MD, USA). Male C57BL/6J and Hmga2^flox/flox^ mice, aged 6–8 weeks and weighing ≈25 ± 2 g, were purchased from the Shanghai Model Organisms Center, Inc. (Shanghai, China). All animals were housed in a temperature‐controlled facility with a 12 h light/dark cycle, maintained at 23 °C with 30–70% relative humidity. No statistically significant differences in age or body weight were observed among the different experimental groups.

### Generation of MCAO‐Induced RI Model

As previously described,^[^ ^5^, ^37^ ^]^ the animals were anesthetized by inhalation of 5% isoflurane in a mixture of 70% nitrous oxide and 30% oxygen, followed by maintenance of anesthesia with 2% isoflurane. Blood oxygen saturation (SpO_2_) was monitored using a pulse oximeter (SurgiVet V3304, Smiths Medical, USA) to ensure SpO_2_ remained above 90%. Body temperature was continuously monitored via a rectal probe and maintained between 36.5 and 37.5 °C using a heating lamp. Subsequently, the skin at the neck incision site was disinfected with an iodine complex, and a midline neck incision was made to expose the right common carotid artery, external carotid artery, and ICA. A silicone‐coated nylon microsurgical filament (L2000; Guangzhou Jialing Biotechnology Co., Ltd., Guangzhou, China) was inserted through the external carotid artery to occlude the right middle cerebral artery. During this procedure, partial whitening of the right pupil was observed in some mice, while the contralateral eye remained normal, indicating successful model induction. In the sham group, all procedural steps were performed as above, except that the mid‐segment of the right carotid artery was not occluded. Following model induction, antibiotic eye drops were administered to prevent infection. Ischemic retinal tissue was collected 24 h after RI for subsequent experiments.

### Primary Analysis of Raw Read Data from snRNA‐Seq

Raw reads were processed to generate gene expression profiles using CeleScope (v1.5.2; Singleron Biotechnologies) with default parameters. Briefly, barcodes and unique molecular identifiers (UMIs) were extracted from R1 reads and subsequently corrected. Adapter sequences and poly‐A tails were trimmed from R2 reads, which were then aligned to the GRCm38 (mm10) transcriptome using STAR (v2.6.1b). Uniquely mapped reads were assigned to exons using the featureCounts function (v2.0.1). Successfully assigned reads sharing the same cell barcode, UMI, and gene were grouped to generate a gene expression matrix for further analysis.

### Quality Control, Dimensionality Reduction, and Clustering

Scanpy (v1.82) was used for quality control, dimensionality reduction, and clustering under Python (v3.7).^[^ ^38^ ^]^ For each sample dataset, the expression matrix was filtered using the following criteria: 1) cells with fewer than 200 genes or within the top 2% of gene counts were excluded; 2) cells within the top 2% of UMI counts were excluded; 3) cells with mitochondrial content >30% were excluded; and 4) genes expressed in fewer than five cells were excluded. After filtering, 21438 cells were retained for downstream analyses, with an average of 887 genes and 1733 UMIs per cell. The raw count matrix was normalized to total counts per cell and logarithmically transformed into a normalized data matrix. The top 2000 variable genes were selected using the flavor = “seurat” setting. Principal component analysis (PCA) was performed on the scaled variable gene matrix, and the top 20 principal components were used for clustering and dimensionality reduction. Batch effects between samples were corrected using Harmony (v1.0) with the top 20 principal components from the PCA.^[^ ^39^ ^]^ Cell clusters were visualized using Uniform Manifold Approximation and Projection (UMAP).

### DEG Analysis

To identify DEGs, the scanpy.tl.rank_genes_groups function was used with default parameters based on the Wilcoxon rank‐sum test. Genes expressed in more than 10% of cells in either of the compared groups and with an average log (fold change) > 0.25 were selected as DEGs. The adjusted p‐value (p_adj) was calculated using the Benjamini–Hochberg correction, and a threshold of 0.05 was applied to determine statistical significance.

### Pathway Enrichment Analysis

GO and KEGG analyses were performed using the “clusterProfiler” R package (v3.16.1).^[^ ^40^ ^]^ Pathways with p_adj < 0.05 were considered significantly enriched. For GSVA‐based pathway enrichment, the average gene expression of each cell type was used as input data.^[^ ^41^ ^]^

### Pseudotime Trajectory Analysis: Monocle2 and RNA Velocity

The differentiation trajectory of monocyte subtypes was reconstructed using Monocle2 (v2.10.0).^[^ ^42^ ^]^ For trajectory construction, the top 2000 highly variable genes were selected using Seurat's (v3.1.2) FindVariableFeatures function, and dimensionality reduction was performed with DDRTree. The trajectory was visualized using the plot_cell_trajectory function in Monocle2. For RNA velocity analysis, the BAM file and reference genome GRCm38 (mm10) were used with Velocyto (v0.2.3) and scVelo (v0.17.17).^[^ ^43^ ^]^

### Intravitreal Injection

Mice were anesthetized via intraperitoneal injection of 1% pentobarbital sodium. Following this, a topical anesthetic was applied using proparacaine hydrochloride eye drops. After allowing the topical agent to take effect for 5 min, the surface fluid of the eyeball was gently removed with a cotton swab. The pupils were dilated with 0.5% compound tropicamide eye drops. Subsequently, the mice were positioned under a surgical microscope. ≈1 mm posterior to the limbus, reagents were injected using a Hamilton microsyringe based on the experimental grouping. The injection needle was maintained within the vitreous cavity for 30 s to ensure proper delivery of the solution. The needle was slowly withdrawn from the eye to avoid damage to the lens and posterior retina. Following this procedure, ofloxacin gel was applied to the right eye to prevent infection.

### Construction and Identification of *Hmga2* Knockout Mice

Homozygous Hmga2^flox/flox^ mice were initially obtained from Shanghai Model Organisms Center, Inc. Genotyping was performed using DNA extracted from mouse tail samples. Intravitreal injections of AAV‐shH10‐CMV‐Cre‐ZsGreen were performed in these homozygous mice, using the Müller cell‐specific serotype (shH10), which effectively targets Müller glial cells and overexpresses Cre recombinase. The control group received an injection of AAV carrying a negative control (NC) in the same eye. After 4 weeks, proteins were extracted from the retinal tissue of the right eye, and WB analysis was performed to confirm the conditional knockout of *Hmga2*, as shown in Figure S1 (Supporting Information).

### H&E Staining

Frozen retinal sections were first immersed in hematoxylin solution for 10 min, followed by rinsing with tap water until a distinct blue coloration was achieved. The sections were then immersed in eosin solution for 2 min and rinsed again with tap water, ensuring the background developed a pink hue. Subsequently, the sections were dehydrated by immersion in a graded ethanol series, from 70% to 100%, with each step lasting 5–10 min to effectively remove moisture. After dehydration, the sections were cleared in xylene and mounted with neutral resin. Retinal thickness was measured in four adjacent areas within a 1 mm radius of the optic disc. Thickness measurements were analyzed and annotated using ImageJ software (v1.8.0; NIH, Bethesda, MD, USA).

### Electroretinogram (ERG) Recordings

Mice were adapted to the dark prior to the experiment to minimize the influence of ambient light on experimental results. In our protocol, compound tropicamide eye drops (5 mg mL^−1^; Shenyang Xingji Co., Ltd., Shenyang, China) were used to dilate the pupils, while ambucaine hydrochloride eye drops (4 mg mL^−1^; Santen Pharmaceutical Co., Ltd., Osaka, Japan) were applied as a corneal surface anesthetic. After intraperitoneal administration with pentobarbital sodium (80 mg kg^−1^), the mice were secured, and ERG recordings were performed following the guidelines of the International Society of Visual Electrophysiology.^[^ ^44^ ^]^ Dark‐adapted ERG responses were recorded using a computer‐based system (MonPack 3; Metrovision, France).

### IF Staining

Following RI induction via MCAO, mice were euthanized under anesthesia at 24 h post‐ischemia. The eyeballs were enucleated and fixed in 4% paraformaldehyde (PFA) for one day, then dehydrated and embedded for cryosectioning. The cryosections were permeabilized using 0.3% Triton X‐100 and blocked with 5% BSA in PBS at 37 °C. Subsequently, primary antibodies corresponding to the experimental protocol were applied and incubated overnight at 4 °C, followed by several PBS washes. The sections were then stained with secondary antibodies for 1 h. Nuclei were counterstained with DAPI (S2110, Solarbio). LC3B‐positive Müller cells were counted in four adjacent areas within 1 mm of the optic disc. Five random fields were selected from each section for cell counting. Quantification was performed by two observers blinded to the group identities and repeated independently at least three times. The following antibodies were used: mouse anti‐glutamine synthetase (GS) (1:100; 20795‐1‐AP; Proteintech); rabbit anti‐LC3B (1:100; 3868; Cell Signaling Technology); rabbit anti‐Hmga2 (1:100; ab97276; Abcam; UK); goat anti‐rabbit IgG (H + L), highly cross‐adsorbed secondary antibody, Alexa Fluor Plus 488 (1:1000; A11034; Invitrogen; USA); and goat anti‐mouse IgG (H + L), highly cross‐adsorbed secondary antibody, Alexa Fluor Plus 555 (1:1000; A32727; Invitrogen, USA).

### Cell Culture

Primary Müller cells were purchased from Shanghai Zhongqiao Xinzhou Biotechnology Co., Ltd. and cultured in complete Müller cell medium supplemented with 10% FBS and 1% penicillin‐streptomycin solution. Once the cells reached a stable state, they were subjected to OGD. Additionally, these cultured primary Müller cells served as the source of Müller cell membranes for the subsequent construction of hybrid fusion membrane carriers for nanomedicine delivery.

Primary RGCs were isolated from postnatal day 3 C57BL/6 mouse pups. The extracted retinas were placed in physiological saline or sterile medium, and the retinal tissue was digested with trypsin for 30 min to facilitate thorough complete dissociation. After digestion, the suspension was filtered through a mesh to remove undigested tissue fragments. The cell pellet was gently washed with culture medium, and the cells were resuspended in appropriate medium, plated onto culture dishes, and maintained in Neurobasal/B27 medium (Invitrogen, Carlsbad, CA, USA).

### In Vitro siRNA Transfection

Cells were seeded in appropriate culture dishes and allowed to grow to 70%–90% confluence, typically 24 h prior to transfection. Hmga2 siRNA was designed and constructed by Hanbio Biotech Co., Ltd. (Shanghai, China). Hmga2 and NC siRNA were transfected into Müller cells using the jet‐PRIME in vitro transfection reagent (Polyplus). The oligonucleotide sequences for Hmga2 siRNA were as follows: sense: 5′‐CCUCUAAAGCAGCCCAGAATT‐3′; and antisense: 5′‐UUCUGGGCUGCUUUAGAGGTT‐3′.

### In Vitro OGD

To mimic ischemic conditions in vivo, the normal medium was replaced with a glucose‐free medium. The primary Müller cells were then incubated at 37 °C in a hypoxic environment composed of 5% CO_2_ and 95% N_2_.

### TUNEL Staining

Samples were fixed with an appropriate fixative (such as 4% PFA) at room temperature for 10–30 minutes at 37 °C and then rinsed with PBS to remove residual fixative (3 times, 5 min each). Permeabilization was performed using 0.1% Triton X‐100 for 5–10 minutes at room temperature. The TUNEL reaction mixture, containing terminal deoxynucleotidyl transferase and labeled dUTP, was then added to the samples and incubated at 37 °C for 1 h.

### WB Analysis

At 24 h after model establishment, the mice were anesthetized and euthanized. The right eyeballs were enucleated, and the retinal tissues were isolated. RIPA lysis buffer was added, and after fragmentation, the lysates were centrifuged, and the supernatants were collected to determine protein concentration. A 12% separation gel was prepared. Electrophoresis buffer was added to the electrophoresis tank, and 30 µg of total protein was loaded per sample. Electrophoresis, membrane transfer, and blocking were performed, followed by incubation with primary antibodies overnight at 4 °C. The next day, the membranes were incubated with goat anti‐rabbit or goat anti‐mouse secondary antibodies (1∶1000), and the protein bands were visualized using a high‐performance imaging system. The gray values of the protein bands were analyzed using the ImageJ software, and the ratio of the gray value of the target bands to that of the internal reference bands was used to represent the relative expression of the target proteins. The primary antibodies used included rabbit anti‐Hmga2 (1:1000; ab97276, Abcam), rabbit anti‐PI3K (1∶1000, AF6241, Affinity Biosciences), rabbit anti‐p‐PI3K (1∶1000, AF3241, Affinity), rabbit anti‐AKT (1∶1000, AF0836, Affinity), rabbit anti‐p‐AKT (1∶1000, AF0832, Affinity), rabbit anti‐Beclin‐1 (1∶1000, AF5128, Affinity), rabbit anti‐LC3B (1:1000; 3868; Cell Signaling Technology), and mouse anti‐GAPDH (1∶10000, AF7021, Affinity).

### Flow Cytometry

After euthanasia, the eyeballs were immediately enucleated and placed in ice‐cold PBS. The retinas were dissected and dissociated into single‐cell suspensions. The resulting primary cells were aliquoted into EP tubes, with each tube containing no fewer than 10^6^ cells, and incubated with Glutamine Synthetase Monoclonal Antibody (MA5‐38531; Thermo Fisher Scientific) under light‐protected conditions on ice for 30 min. FACS was performed to isolate Müller cells using the BD FACSAria IIu cell sorter.

The sorted RGC single‐cell suspension was centrifuged at 500 × *g* for 5 min, and the supernatant was discarded. The cells were washed three times with 2 mL PBS and centrifuged again at 500 × *g* for 5 min. After discarding the supernatant, Annexin V and propidium iodide (PI) staining solutions were added, and the cells were incubated in the dark for 15 min. Subsequently, 200 µL PBS was added for resuspension, and samples were immediately analyzed using the EXPO32 ADC XL4 Color flow cytometer. The results were processed with EXPO32 ADC Analysis software.

### Mouse Cytokine Array

As previously described, the supernatant was collected from cultured Müller cells across different experimental groups, and a mouse cytokine array was performed according to the manufacturer's instructions (QAM‐CAA‐1000; RayBiotech, USA).

### ELISA

ELISA was performed according to the manufacturer's instructions. Briefly, a series of gradient dilutions of protein standards was prepared, and absorbance at 450 nm was measured to generate a standard curve. In cellular experiments, the culture medium from Müller cells was centrifuged at 2000–3000 rpm for 30 min. After centrifugation, the supernatant was transferred to a sterile tube for subsequent analysis. In vivo experiments involved collecting mouse retinas from each group at specified time points post‐ischemia for further detection. The following ELISA kits were used: Mouse IL‐1β ELISA Kit (ab197742; Abcam), Mouse TNFα ELISA Kit (ab108910; Abcam), Mouse IL‐2 ELISA Kit (ab100706; Abcam), Mouse IL‐4 ELISA Kit (ab100710; Abcam), Mouse IL‐5 ELISA Kit (ab100711; Abcam), and Mouse TARC (CCL17) ELISA Kit (ab171337; Abcam).

### TEM

Collected Müller cells were fixed in 4% glutaraldehyde and stored at 4 °C. The prepared samples were placed on specialized metal grids for TEM. By adjusting the optical system and the sample position, the electron beam was precisely directed through the center of the sample, and the imaging system was calibrated to obtain a clear image.

### Autophagic Flux Detection

Müller cells were transduced with an adenovirus expressing HBAD‐mRFP‐GFP‐LC3 (Hanbio) to assess autophagic flux. The reduction in GFP fluorescence indicates the fusion of lysosomes with autophagosomes to form autolysosomes. Given that GFP was sensitive to acidic conditions, its fluorescence was quenched upon lysosome–autophagosome fusion, allowing only red fluorescence to be detected. Yellow puncta represent autophagosomes, while red puncta indicate autolysosomes.

### Co‐IP

Cell samples were collected and lysed with RIPA buffer on ice for 30 min to ensure complete protein extraction. Cellular debris was removed via centrifugation at 12000 rpm for 5–10 minutes, and the supernatant was collected, while the pellet was discarded. The supernatant was then incubated with agarose beads lacking bound antibody (sc‐2003, Santa Cruz Biotechnology, Inc.) under gentle rotation at 4 °C for 1 h to eliminate nonspecifically bound proteins. Subsequently, either a specific primary antibody or 1 µg of IgG was added to the supernatant and incubated overnight at 4 °C. The immunoprecipitates were then analyzed via immunoblotting. The antibodies used included rabbit anti‐Hmga2 (1:50; ab97276, Abcam) and rabbit anti‐PI3K (1:50, AF6241, Affinity).

### Molecular Docking

The predicted structures of PI3K and HMGA2 were generated using AlphaFold software. To ensure accurate docking results, the protein was prepared using AutoDockTools (v1.5.7): water molecules were manually removed and polar hydrogens were added. The GRAMM docking web server was used for protein–protein docking. The resulting protein–protein complex was further manually optimized by again removing water molecules and adding polar hydrogen using AutoDockTools. Protein–protein interactions were predicted, and visualizations were generated using PyMOL. GRAMM's scoring system evaluates conformational plausibility by integrating energy terms such as molecular shape complementarity, electrostatic interactions, hydrogen bonding potential, and hydrophobic effect. The score reflects the stability of the predicted conformation through a combination of empirical parameters and physical force fields. Typically, a higher score indicates a more thermodynamically and geometrically stable conformation.^[^ ^45^ ^]^ Molecular dynamics simulations were then conducted to calculate the binding free energy of the complex formed between HMGA2 and the ligand P85A using the Molecular Mechanics/Generalized Born Surface Area method. This approach assessed thermodynamic stability and identified key energetic contributors to the interaction. Binding free energy was calculated without including entropy terms.

### Synthesis of siRNA‐Hmga2@LMM

Soybean phosphatidylcholine, 1,2‐dioleoyl‐3‐trimethylammonium‐propane chloride, dimyristoyl glycerol‐polyethylene glycol 2000, and cholesterol (in a mass ratio of 40%:20%:15%:15%; corresponding to 0.4, 0.2, 0.15, and 0.15 mg, respectively) were dissolved in 3 mL of anhydrous ethanol and transferred to a round‐bottom flask. siRNA (10%; 0.1 mg) was dissolved in 1 mL of citrate buffer (50 mm citrate, pH 4.0) containing 25% ethanol, then slowly added to the lipid solution, mixed thoroughly, and incubated for 20 min. Diethyl pyrocarbonate‐treated water was added, and the mixture was subjected to ultrasonication and passed through a liposome extruder fitted with a 200 nm filter membrane. Nanodialysis (using a polycarbonate membrane with a pore size of 10 nm) was used to remove free molecules, salts, and ethanol from the solution, yielding siRNA‐Hmga2@Lip. Mouse Müller cell membranes were then added to the liposomes at a mass ratio of 1:1, thoroughly mixed, and sonicated in a water bath to facilitate fusion of the liposomes with the cell membranes until the solution appeared clear, resulting in siRNA‐Hmga2@LMM. The hydrodynamic diameter, ζ potential, and stability of the nanomaterials were measured using a Brookhaven NanoBrook 90Plus PALS. Nanoparticle tracking analysis of LMM was performed using the NanoSight NS300 system.

### Statistical Analysis

Statistical analysis was performed using GraphPad Prism (v9.0). All data are representative of three independent experiments. Continuous variables were expressed as mean ± standard deviation of the mean for normally distributed data. One‐way or two‐way analysis of variance was used, depending on the experimental design. A p‐value of < 0.05 was considered statistically significant.

## Conflict of Interest

The authors declare no conflict of interest.

## Supporting information



Supporting Information

## Data Availability

The data that support the findings of this study are available from the corresponding author upon reasonable request.
