# Supplementary material for: Single‐Cell Transcriptome Analysis Reveals That Hmga2 Regulates Neuroinflammation and Retinal Function by Modulating Müller Cell Autophagy Through PI3K/AKT Signaling Following MCAO‐Induced Retinal Ischemia
Source: Adv Sci (Weinh). 2025 Aug 30;12(43):e02534. doi: 10.1002/advs.202502534 (PMC12631878; doi:10.1002/advs.202502534)
Supplement: Supplementary file 1 — Supporting Information [file ADVS-12-e02534-s001.docx]

Supporting Information

**Single-cell transcriptome analysis reveals that Hmga2 regulates neuroinflammation and retinal function by modulating Müller cell autophagy through PI3K/AKT signaling following MCAO-induced retinal ischemia**

*Weihao Lv^1^*, Juzheng Yuan^3^*, Zhe Ruan^4^*, Ya-nan Dou^1^*, Hongchen Zhang^1^, Xiaowei Fei^1^, Leiying Chen^2^, Zehan Zhang^1^, Kai Yang^5^, Xiuquan Wu^1#^, Zhou Fei^1#^, Fei Fei^2#^*

**Supplementary method**

**DNA pull down-MS**

DNA pull down-MS is an effective method for studying the interaction between DNA and proteins in vitro, which can explore the interaction between DNA and transcriptional regulatory proteins. The procedure is as follows: A specific biotin-labeled probe is designed based on the target DNA region and immobilized onto streptavidin-coated magnetic beads. Nuclear extracts are incubated with the bead-bound DNA probe, allowing proteins to be specifically captured by the probe. Protein complexes bound to the target DNA fragment are purified. The bound proteins are identified using mass spectrometry (MS). For data analysis in this experiment: Both peptide-level and protein-level False Discovery Rates (FDR) were controlled at 0.01. Common contaminant proteins (e.g., keratins) were first removed. Quantitative analysis was then performed to identify specifically bound proteins by comparing the experimental group (Test) to the negative control group (Ctrl). This analysis was based on Fold Change (FC) values and the number of unique peptides, enabling the subtraction of background signals from the control.

**Plasmid transfection**

Plasmids were designed by Hanbio Biotechnology Co., Ltd. 293T Cell and Müller cells were transfected with plasmids by using jetPRIME Buffer (#201000003; Polyplus) reagent to stably over express Max (pLV-m-max). The plasmid transfection process was strictly according to the protocol manual.

**Biolayer interferometry (BLI)**

The binding affinity of HMGA2 protein with PIK3R1 protein were determined using the GatorPlus biolayer interferometry system (Gator Bio, Palo Alto, CA, USA). All steps were performed at 30 ℃, 1000 rpm. Briefly, Anti-His sensors (Gator Bio) were dipped into the Hmga2 protein (0.5 μg/ml) solutions for 30 s for loading and then dipped into various concentrations of PI3K (0-3 μM) in PBS, 0.05% Tween 20 buffer. A duplicate set of sensors was used as background binding controls that were incubated in a buffer without proteins. All the data were analyzed by Gator Bio data analysis software. The equilibrium dissociation constant (KD) values were calculated from the ratio of Koff to Kon, based on global fitting of several curves generated from serial dilutions of the PI3K protein.


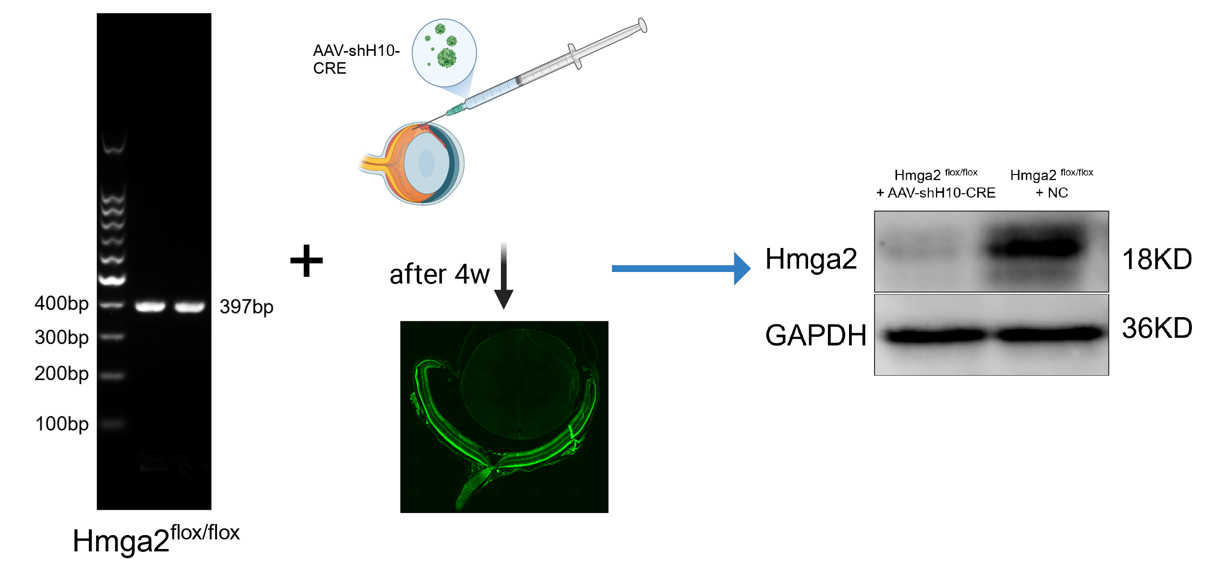


**Figure S1.** Generation of conditional knockout mice of Müller cell *Hmga2* gene in mouse retina


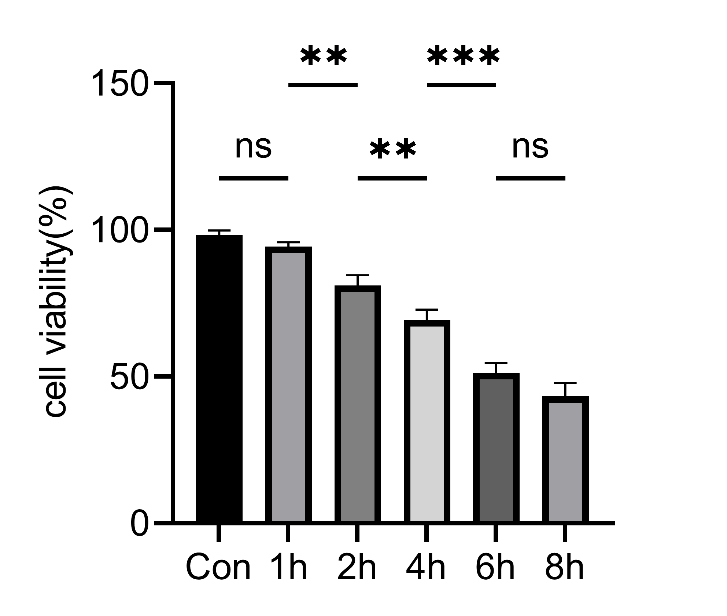


**Figure S2.** The changing trend in Müller cell activity after different OGD time points. After 6 h of OGD, the activity of Müller cells decreased to 50%.


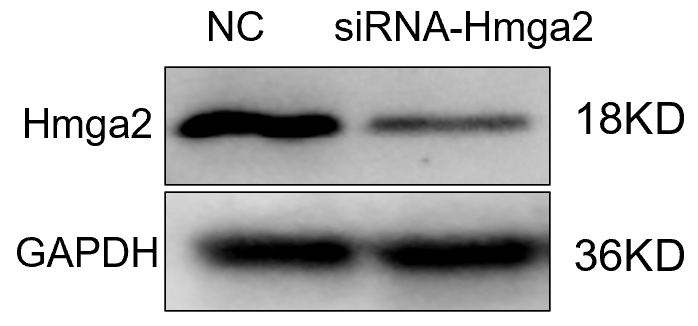


**Figure S3.** Verification of knockdown effect of siRNA-Hmga2 in Müller cells.


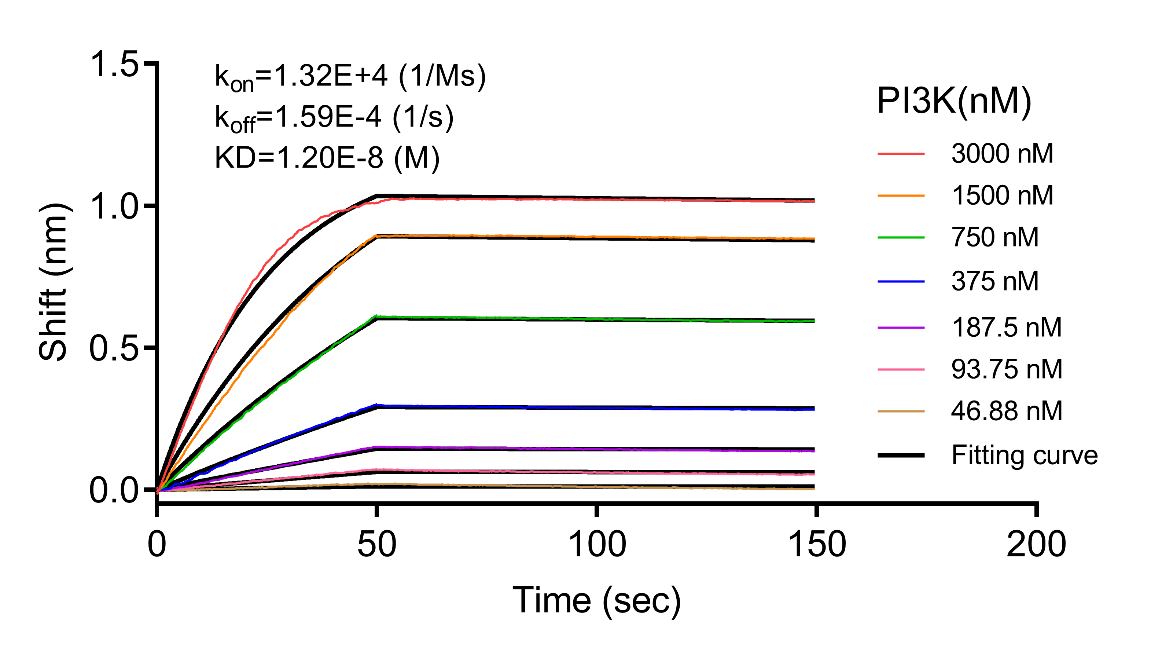


**Figure S4.** BLI binding analysis of PI3K to immobilized Hmga2. The concentrations of PI3K used for each kinetic measurement are indicated. Representative data are shown in different color, with fitted (1:1 Langmuir binding models) in black. The KD values are shown.


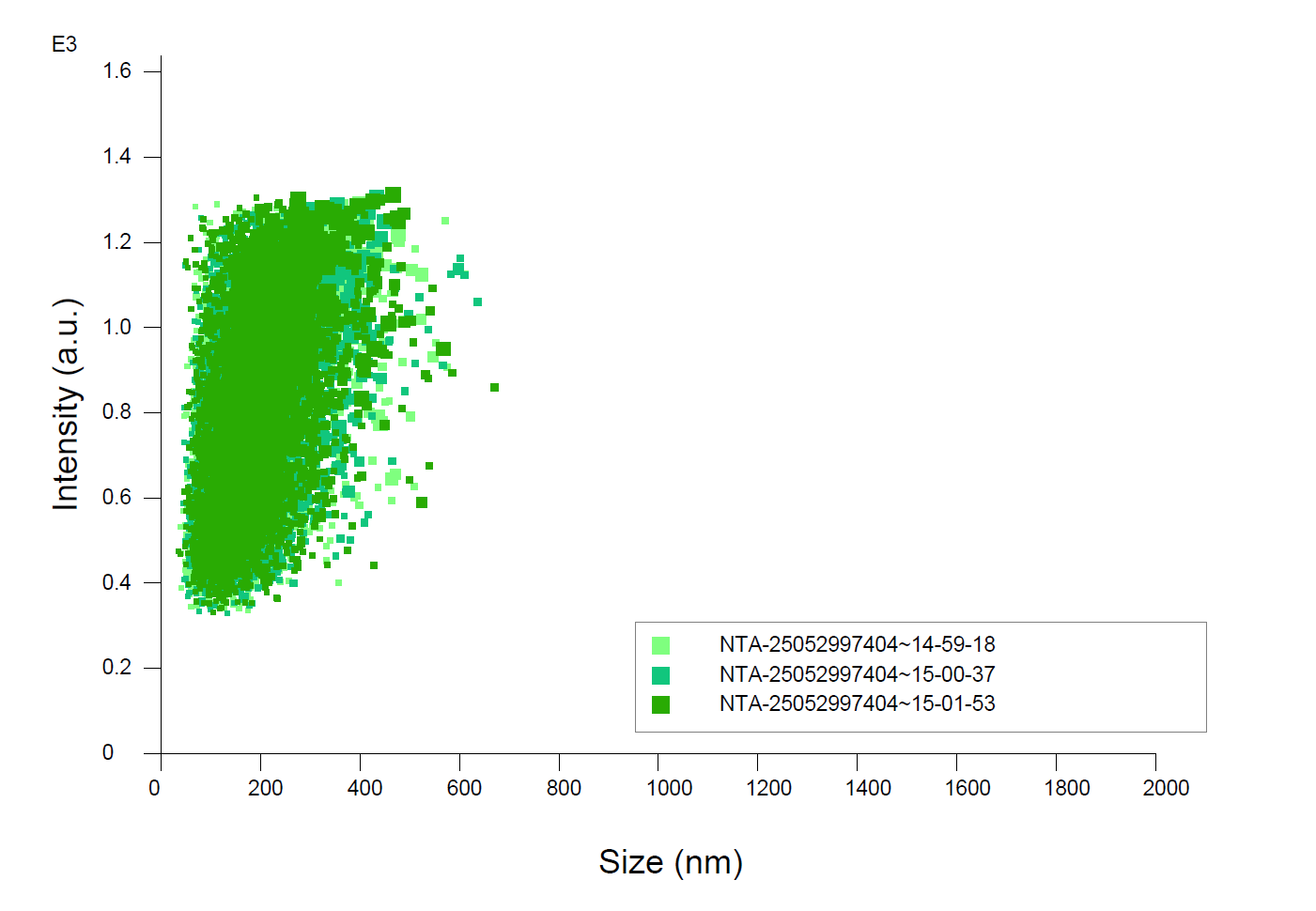


**Figure S5.** Scatter plot of particle size and strength distribution of hybrid drug siRNA-Hmga2@LMM.


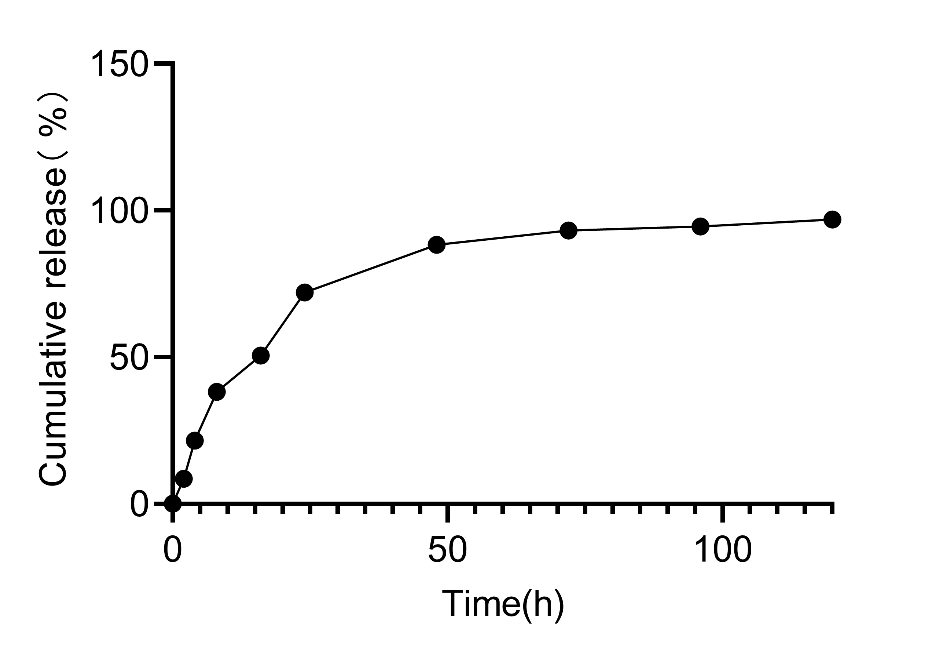


**Figure S6.** Cumulative release of hybrid drug siRNA-Hmga2@LMM.

**Highly Expressed Hmga2 is Regulated by Transcription Factors of the Max**

Using DNA pull-down MS technology, it was identified 208 proteins bound to the Hmga2 gene. To screen for potential transcription factors (TFs) from these candidates, we first compiled a comprehensive list of mouse TFs by screening the TRRUST and Animal TFDB v4.0 databases. Subsequently, we input the promoter sequence of the Hmga2 gene into Animal TFDB v4.0 for prediction analysis and performed an intersection analysis between these prediction results, the 208 experimentally identified binding proteins, and the TF lists from both TRRUST and Animal TFDB v4.0, ultimately identifying the six most likely candidate transcription factors. The six transcription factors were ranked according to their fold change (FC), with Max found to exhibit the largest FC (Figure S7A). Based on this observation, it was hypothesized that Max potentially functions as a transcription factor binding to the Hmga2 promoter to regulate its expression. To test this hypothesis, a Max overexpression plasmid was constructed and transfected into cells, and Hmga2 expression was assessed via western blotting. Hmga2 expression was found to be positively regulated by Max in both 293T cells and primary Müller cells (Figure S7B-G). Compared with that of the Con group, the expression level of Max in the Müller cells in the OGD group was significantly increased (Figure S7H, I).


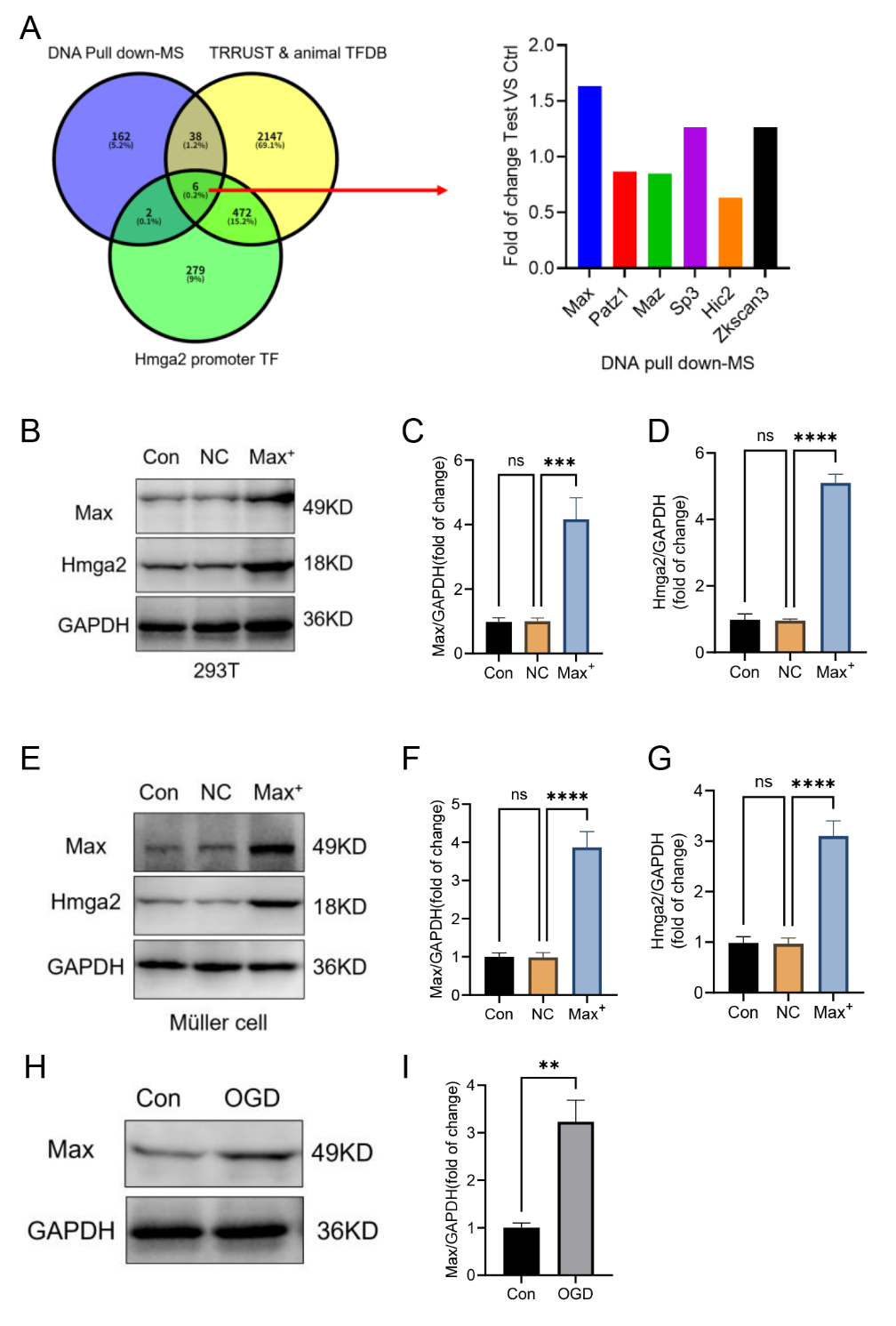


**Figure S7.** The expression of Hmga2 was regulated by the transcription factor Max. **A** The six most likely transcription factors obtained by DNA down-pull MS experimental data combined with TRRUST, animal TFDB database screening was ranked according to FC (Test VS Ctrl). **B** Western blot was performed to detect the regulatory effect of Max expressions on Hmga2 protein in 293T cell. **C-D** Quantification of result in B. **E** Western blot was performed to detect the regulatory effect of Max expressions on Hmga2 protein in primary Müller cells. **F-G** Quantification of result in E. **H** Expression levels of Max in the Müller cells of each group in vitro. For panel (C, D, F, G, I): ns, no significance, ** p < 0.01, *** p < 0.001, **** p < 0.0001 by one-way analysis of variance or Student’s *t* test. All data are representative of three independent experiments.
